# Wnt signaling in triple negative breast cancer is associated with metastasis

**DOI:** 10.1186/1471-2407-13-537

**Published:** 2013-11-10

**Authors:** Nandini Dey, Benjamin G Barwick, Carlos S Moreno, Maja Ordanic-Kodani, Zhengjia Chen, Gabriella Oprea-Ilies, Weining Tang, Charles Catzavelos, Kimberly F Kerstann, George W Sledge, Mark Abramovitz, Mark Bouzyk, Pradip De, Brian R Leyland-Jones

**Affiliations:** 1 Edith Sanford Breast Cancer, Sanford Research, 2301 E 60th Street N, Sioux Falls, SD 57104, USA; 2 Department of Internal Medicine, University of South Dakota, Vermillion, SD 57069, USA; 3 AKESOgen, Inc., Atlanta, GA 30071, USA; 4 Department of Pathology and Laboratory Medicine, School of Medicine, Emory University, Atlanta, GA 30322, USA; 5 Winship Cancer Institute, Atlanta, GA 30322, USA; 6 Department of Biostatistics and Bioinformatics, Rollins School of Public Health, Emory University, Atlanta, GA 30022, USA; 7 Department of Pathology, St. Mary’s Hospital, McGill University, Montreal, QC H3A 1G5, Canada; 8 Indiana University Cancer Center, Indiana Cancer Pavilion, Indianapolis, IN 46202, USA; 9 VM Institute of Research, Montréal, QC H3G 1L5, Canada; 10 Current address: Centers for Disease Control and Prevention, 1600 Clifton Road, N.E., Atlanta, GA 30333, USA; 11 Current address: Director of Pathology, Ville Marie Multidisciplinary Medical Centre, 1538, Sherbrooke Street W., Montréal, QC H3G 1L5, Canada; 12 Current address: CHU Sainte-Justine Research Centre, Montréal, QC H3T 1C5, Canada; 13 Department of Pathology, Emory University School of Medicine, Atlanta, GA 30322, USA

**Keywords:** Breast cancer, Triple negative, Wnt, FFPE, Microarray

## Abstract

**Background:**

Triple Negative subset of (TN) Breast Cancers (BC), a close associate of the basal-like subtype (with limited discordance) is an aggressive form of the disease which convey unpredictable, and poor prognosis due to limited treatment options and lack of proven effective targeted therapies.

**Methods:**

We conducted an expression study of 240 formalin-fixed, paraffin-embedded (FFPE) primary biopsies from two cohorts, including 130 TN tumors, to identify molecular mechanisms of TN disease.

**Results:**

The annotation of differentially expressed genes in TN tumors contained an overrepresentation of canonical Wnt signaling components in our cohort and others. These observations were supported by upregulation of experimentally induced oncogenic Wnt/β-catenin genes in TN tumors, recapitulated using targets induced by Wnt3A. A functional blockade of Wnt/β-catenin pathway by either a pharmacological Wnt-antagonist, WntC59, sulidac sulfide, or β-catenin (functional read out of Wnt/β-catenin pathway) SiRNA mediated genetic manipulation demonstrated that a functional perturbation of the pathway is causal to the metastasis- associated phenotypes including fibronectin-directed migration, F-actin organization, and invasion in TNBC cells. A classifier, trained on microarray data from β-catenin transfected mammary cells, identified a disproportionate number of TNBC breast tumors as compared to other breast cancer subtypes in a meta-analysis of 11 studies and 1,878 breast cancer patients, including the two cohorts published here. Patients identified by the Wnt/β-catenin classifier had a greater risk of lung and brain, but not bone metastases.

**Conclusion:**

These data implicate transcriptional Wnt signaling as a hallmark of TNBC disease associated with specific metastatic pathways.

## Background

Stratification of breast cancer (BC) into distinct histological or molecular subtypes has clinical utility for prognosis of outcome and prediction of treatment [1]. Breast cancer subtypes based on clinical or molecular characteristics are typically referred to as hormone-receptor positive (HR+) or luminal, HER2-amplified, and triple negative (TN) or basal-like. TNBC are defined by negative of expression of ER, PR, and HER2 amplification and are associated with a higher grade, undifferentiated metaplastic histology, stem cell-like characteristics, invasiveness, higher metastatic potential, and inconsistently effective therapies. Triple negative and basal-like subtypes have a significant overlap, and lack standardized clinical markers that differentiate the two subtypes, something that underlines the inherent heterogeneous nature of these subtypes [2-4]. Triple negative and basal-like subtypes portend some of the worst prognoses in BC [5,6], and have the most challenging diagnosis among BC patients due to the potential aggressive nature of the disease and limited number of therapeutic options available. Studies of Shah et al. indicated that understanding the biology and therapeutic responses of patients with TNBC will require the determination of individual tumor clonal genotypes [7]. Despite of making considerable progress in cancer research, the mortality rate of TNBC has remained unchanged in the last decade primarily due to the lack of specific target identification. The allure of the emerging genomic technologies in cancer in their ability to generate new biomarkers that predict how individual patients will respond to various treatments has not been completely successful in TNBC. Only recently, the first comprehensive genomic analysis of a basal-like breast cancer was performed by using massively parallel sequencing technology [8]. Despite a few recent reports that indicated the involvement of certain genes/signaling molecules related to tumorigenic pathways [4,9-11] in these subsets, there remains an unmet need for an in-depth study to identify driver pathways in these closely associated subtypes of BC.

We analyzed mRNA expression from archival formalin-fixed paraffin-embedded (FFPE) tumor specimens from two breast cancer cohorts. Our data, together with meta-analysis of previous breast cancer microarray studies, indicate Wnt pathway activation in TNBC subtypes and provides evidence for an increased Wnt/β-catenin signaling associated with high grade, poor prognosis, and metastatic disease. In light of reports from Reis-Filho’s team [12], who established a preferential increase in β-catenin protein (immunohistochemistry) in TNBC patients, our study not only identifies clinical markers associated with Wnt signaling such as histological grade 3 tumors and triple negative pathological subtype, but also indicated an upregulated state of Wnt signaling increasing the risk for brain and lung metastases, thus classifying Wnt signaling as a rational target in TNBC. The inhibition of metastasis-associated phenotypes, integrin-directed migration and invasion following Wnt-antagogist, WntC59 as well as β-catenin SiRNA provided mechanistic proof of concept for the involvement of this pathway in the progression of the disease and its clinical outcome.

## Methods

### Study cohorts

Archived FFPE tumor specimens were obtained from St. Mary’s Hospital, Montreal, Quebec, Canada (Quebec cohort) and Grady Memorial Hospital, Atlanta, GA (Georgia cohort) according to institutional guidelines (Emory University). Tumor content (> 50% inclusion criteria) was assessed by a board certified pathologist. Cohort sizes of 107 and 166 patients from St. Mary’s and Grady Hospitals respectively were analyzed at the Emory Biomarker Service Center (Winship Cancer Institute, Atlanta, GA). Both Georgia and Quebec samples (FFPE) were acquired following the acceptance of our protocols by the ethics committees (Emory University, USA for Georgia study and Canada for Quebec study) of the respective institutions. Archived FFPE samples were used for the study. All archived FFPE tumour specimens were obtained from St Mary’s Hospital (Montreal, Quebec, Canada) according to institutional guidelines.

Data Deposition: Microarray data curetted under GEO series accession numbers [GSE17650 & GSE18539].

### RNA preparation, quality control, and DASL assay

FFPE samples were analyzed using DASL (cDNA-mediated, Annealing, Selection, Extension, and Ligation) expression chemistry (Illumina, Inc., San Diego, CA), on the Illumina human cancer panel and a custom panel with breast cancer related genes. Depending upon tissue availability, specimens were obtained via either three 5 μm sections or 0.5 mm cores. Deparaffinization, RNA extraction and RNA purification were carried out using commercially available RNA High Pure Kit (Roche, Mannheim, Germany) modified as previously described [13]. Prior to DASL analysis, RNA quality was assessed via RPL13a TaqMan assay using a threshold Ct of less than 29.5. For samples that passed QC, 200 ng of total RNA was used as input for the DASL assay according to the manufacturer’s protocol (Illumina, San Diego, CA). When ample RNA was available, RNA replicates were run to test for reproducibility of the DASL assay.

To determine whether the DASL assay yields comparable data to IHC data, the DASL assay gene intensity (expression) data were compared with the available IHC protein expression data for ER, PR and HER2 on the set of tumor samples. Standard clinical IHC testing was conducted for ER, PR and HER2 according to guidelines based on ASCO/CAP. This is now stated in the Methods section (p. 7, last sentence). “In total, we obtained 87 FFPE breast carcinomas that had previously been scored for the breast cancer markers, ER, PR and HER2 by immunohistochemistry (IHC) according to guidelines based on the ASCO/CAP recommendations for ER, PR and HER2 testing (ER/PR testing [http://www.cap.org/apps/docs/laboratory_accreditation/summary_of_recommendations.pdf]; HER2 testing [http://www.cap.org/apps/docs/committees/immunohistochemistry/summary_of_recommendations.pdf])”. Once standardized, the similar protocol was followed in both the cohorts. The comparison of IHC data with DASL data for ER, PR and HER2 had been carried out as mentioned in our earlier publication. Our data show that the concordance of DASL data with IHC data for all three receptors is very high, which is consistent with our previous published work relating mRNA and IHC protein levels [14]. Once standardized, the similar protocol was followed in both the cohorts. A detailed list of the gene-composition of the Illumina human cancer panel, and the breast cancer related genes has been published earlier by our group [14]. We have added a supplementary table with the gene-list (Additional file 1: Table S1, Additional file 2: Table S2) to the revised-MS for the convenience of the reader. The detailed procedure to test the reproducibility of the DASL assay has been already published by our group [14] (The specific details are noted in the Additional file 3 of the above mentioned published article). We used 80 replicates for the standard panel, and 100 replicates for breast cancer custom panel.

### Biochemical analyses

Western blots were performed by solubilizing cell lines with lysis buffer (150 mM NaCl, 6 mM Na2HPO4, 4 mM NaH2PO4, 2 mM EDTA, 1% sodium deoxycholate, 1% NP-40, 0.1% SDS, 1% aprotinin, 0.2 M sodium orthovanadate, and 0.1 M phenylarsineoxide), and lysates were assayed for total protein (Bio-Rad protein assay kit) using BSA as a standard. The normalized lysates (20–40 μg protein) were resolved by 12.5% SDS-PAGE and transferred to nitrocellulose membranes [15]. Membranes were probed with anti-β-catenin (Abcam Inc, Cambridge, MA) antibody, and visualized with enhanced chemiluminescence reagent combined with peroxidase-conjugated IgG [16].

### *In vitro* knockdown of β-catenin protein by SiRNA

Breast cancer cell line (MDA-MB231) was seeded onto 6-well tissue culture dishes, and allowed to attach in culture medium supplemented with 10% FBS. A cell density of 60% to 70% was used for the transient transfection (Lipofectamine 2000) of β-catenin-specific SiRNA (Invitrogen, NY; CTNNB1 VHS50819) into MDA-MB231 cells according to the manufacturer’s instructions. Transfected cells were collected after 24, 48, and 72 hours for analyses [16].

### TCF/LEF promoter activity assay

A luciferase-based reporter gene was used to measure promoter activity of the TCF/LEF transcription factor [17]. For SiRNA based study, cells were transiently transfected with beta-catenin SiRNA [18]. After beta-catenin siRNA transfection for 24 hours, the cells were transiently transfected with the reporter construct TOPflash or FOPflash. In brief, cells were co-transfected with 2.5 μg TOP flash, a synthetic luciferase-based promoter plasmid (sensitive to the activity of the β-catenin/ TCF-4 complex, containing three copies of the TCF-4 binding site upstream of a firefly luciferase reporter gene) using the Lipofectamine 2000. In the other set of cells, an equal amount of the mutant form of the above promoter (FOP flash) was co-transfected using the same transfection reagent. FOPflash has mutated copies of Tcf/Lef sites and is used as a control for measuring nonspecific activation of the reporter. Twenty hours after TOPflash or FOPflash transfection, luciferase assay was performed. Relative luciferase activity (in arbitrary units) was reported. In a separate set of experiments, cells were co-transfected either with TOP flash or FOPflash using lipofectamine. After 12 hour incubation, each set was treated with sulindac sulfide for 24 hours. The relative luciferase activity (TOP flash/FOP flash) was calculated from triplicate experiments.

### Cell line based phenotypic assays

Fibronectin directed migration assay was performed on Wnt-antagonist, WntC59 (Cellagen Technology, LLC, San Diego, CA) treated or β-catenin SiRNA transfected MDA-MB231 cells by transwell assay and scratch assay. Invasion assay was performed by transwell assay. Haptotaxis assays were carried out using transwell migration chambers (Costar Corp.) as previously described [16]. Cells were added into the upper chamber of the transwell containing the through which they were allowed to migrate over time to the fibronectin-coated side. Control experiments involved coating both sides of the membrane with fibronectin. *In vitro* wound healing assays were performed as previously described [16]. In brief, after coating plates with fibronectin, wounds were created by scratching the confluent monolayer of cells. The width of the “scratched” area was measured from randomly chosen fields using either Olympus DP72 system or Axiovert 200 M, Zeiss system. Student’s t test was used to determine the statistical significance.

### Confocal microscopy and real-time video microscopy of live cells

To study the cytoskeletal arrangement, HCC38 and MDA-MB231TNBC cells were fixed, and permeabilized with PHEMO buffer. Phalloidin 555 was used for staining the cytoskeleton filamentous-actin and DAPI as a counter stain. Cells were imaged using a Zeiss (Thornwood, NY) LSM 510 Meta confocal microscope with a Plan-Apochromat oil objective. Images were acquired using Zeiss LSM 510 software and processed using Adobe Photoshop CS3. To study the involvement of Wnt-pathway in integrin-directed migration in real time, video microscopy was performed. A scratch-would healing assay was performed on the confluent layer of cells (grown on fibronectin-coated glass-cover slip culture- dishes; Mattek, Ashland, MA). Time-lapse images were acquired with a Perkin Elmer Ultraview ERS (Norwalk, CT) disk-spinning confocal system, mounted on a Zeiss Axiovert 200 M inverted microscope equipped with a 37°C stage warmer, incubator, and humidified CO2 perfusion system. Bright-field images were acquired with a Hamamatsu Orca-ER camera with a Plan-Neoflour 10× objective (NA 0.75; 1×1 binning) at 10 minutes intervals for each image set.

### Data and statistical analysis

A full description can be found in the supplementary methods. In brief, DASL transcript intensities were quantile normalized in GenomeStudio and replicates were mean combined. Differential transcripts were determined using permutation testing [19] with a false discovery rate (FDR) less than 1% and a 1.5 fold-change. Hierarchical clustering was performed using the “heatmap.2” function of the R/Bioconductor package “gplots” [20]. KEGG signal transduction pathways [21] were analyzed for overrepresentation in the triple negative subtype using Fisher’s exact test and pathway expression was determined using the mean of normalized pathway components. Differential pathway regulation was assessed by a t-test with Bonferroni’s correction applied and permutation testing. HMEC oncogenic data [22] was downloaded from Gene Expression Omnibus (GEO) [23] and pathway expressions were calculated as mean of differentially expressed genes with induced genes weighted positively and inhibited genes negatively. Analysis of Wnt regulation induced by Wnt3A from Nguyen et al. [24] employed the same methods for the LWS-81 genes. Meta-analysis data [25-32] were downloaded from GEO [23] and Affymetrix CEL files were MAS 5.0 normalized with a target intensity of 600. Agilent normalized series matrix files were downloaded and duplicate samples between studies were removed. A nearest shrunken centroid classifier implemented in the R/Bioconductor package “pamr” [33] was trained on β-catenin induced data from Bild et al. [22] and applied to 11 studies and 1,878 patients. Overrepresented pathological subtype, intrinsic subtype, histological grade, and lymph node status in Wnt + (Wnt classifier signature) patients were analyzed using Fisher’s exact test and Kaplan-Meier survival curves were created in R/Bioconductor using the “survival” package with significant differences in risk calculated using the log-rank test. The details of the analyses and statistical methods are presented in the supplementary section as “Additional file 4”.

## Results

### Gene expression was reproducible and concordant with clinical pathological subtype

Messenger RNA expression from FFPE samples were characterized using cDNA-mediated, Annealing, Selection, Extension, and Ligation (DASL) [34] for two populations of breast cancer patients: one from Quebec, and the other from Georgia. Quebec samples came from an unselected patient population from a community hospital in Canada whereas the Georgia cohort was preferentially selected for TNBC. The Quebec cohort was representative of the breast cancer population at the local hospital, whereas the Georgia cohort was preferentially selected for TNBC (Additional file 3: Figure S1H, K and L). Other inclusion criteria required specimens with more than 50% tumor content, matching pathology records for ER, PR, and HER2, and at least 200 ng of RNA. RNA was extracted and further quality controlled as previously described [13]. Samples were run on two DASL panels; one a commercially available panel targeted at cancer related genes, the other a panel targeting genes relevant to breast cancer. RNA was run on both DASL panels with technical replicates when sufficient RNA was available, and had an average Pearson correlation coefficient (R2) of 0.96. Common genes (152) between the two DASL panels measured a correlation (R2) of 0.88 (Additional file 3: Figure S1A-F). Expression of ESR1 (transcript for ER), PGR (transcript for PR), and ERBB2 (transcript for HER2) corresponded well to pathology immunohistochemistry (IHC) records. DASL expression of ESR1 and PGR were greater in HR + as compared to TNBC and HER2+ subtypes, and likewise ERBB2 expression was higher in HER2+ samples compared to TNBC and HR + subtypes in all cohorts (Additional file 3: Figure S1G-L). These results are reproducible and consistent with pathology records.

### Wnt signaling is upregulated in TNBC

**Figure 1:**
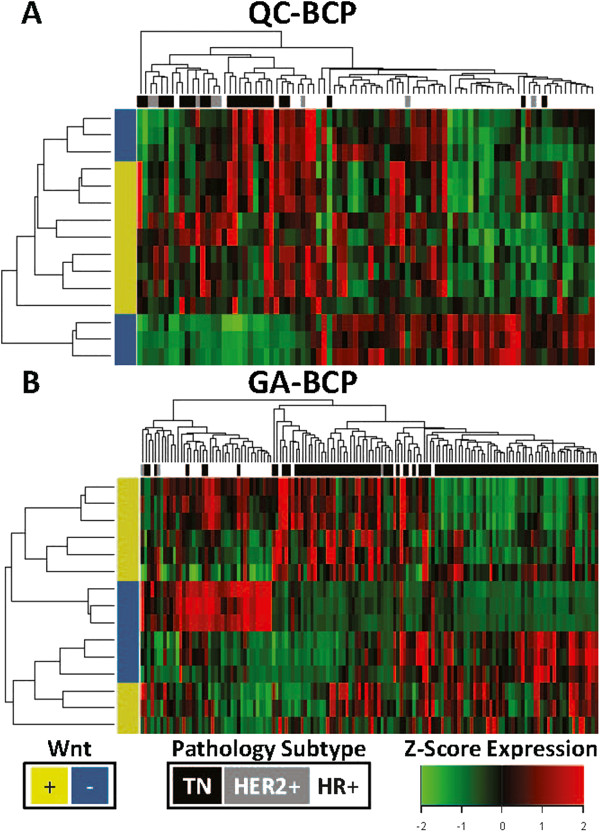
**Unsupervised hierarchical clustering of Wnt3A induced genes from the LWS-81 signature (shown in the figure as Wnt+) **[24]** common to the Breast Cancer DASL panel segregated triple negative tumors (shown in black) from other subtypes in both A) Quebec (QC-BCP) and B) Georgia (GA-BCP) cohorts.** Each row represents a transcript, and each column represents a tumor sample, with triple negative denoted in black, HER2+ in grey, and HR + in white. Yellow indicates genes upregulated in response to Wnt3A and blue indicates genes down regulated. High expression is represented by red and low expression by green.

**Figure 2:**
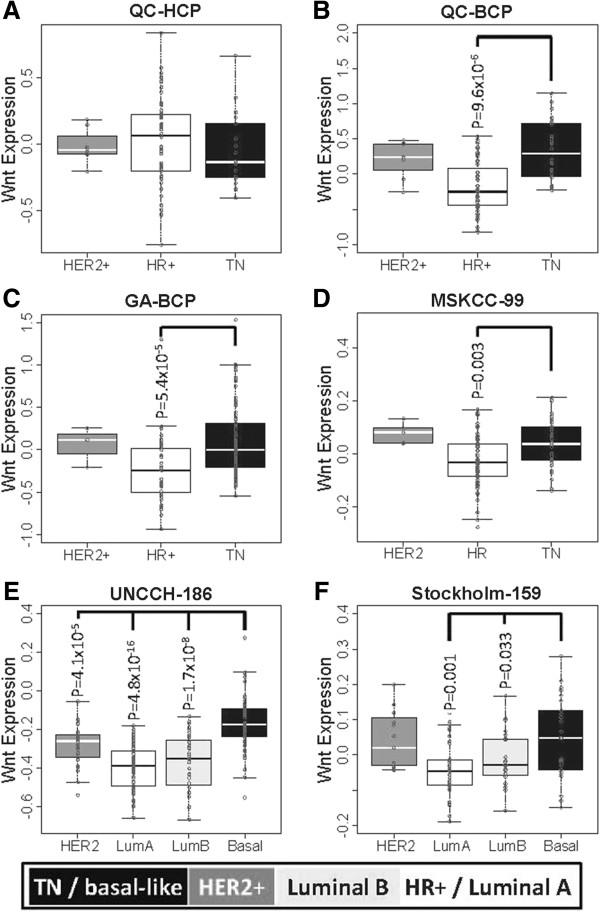
**Lung cancer Wnt induced (LWS-81) genes **[24]** used to assess Wnt transcriptional regulation in the Quebec cohort profiled on the A) human cancer panel (QC-HCP) and B) breast cancer panel (QC-BCP) as well as C) Georgia (GABCP), D) MSKCC-99 **[29]**, E) UNCCH-186 **[30]**, and F) Stockholm-159 (STH-159) cohorts **[31]**.** Significant p-values after Bonferroni’s correction are shown relative to the TNBC subtypes.

Differentially expressed transcripts in the TNBC subtype of the Quebec and Georgia cohorts were annotated for canonical signal transduction pathways using the KEGG database [21]. After Bonferroni correction for multiple hypotheses testing, the only significant result was the Wnt signaling pathway in the Quebec cohort (p = 0.048, Fisher’s exact test; (Additional file 3: Figure S2A-C). Further analysis of three published microarray studies, including a 99 patient cohort with pathology subtype from Memorial Sloan-Kettering Cancer Center (hereafter MSKCC-99) [35], a 186 patient cohort with intrinsic subtype from University of North Carolina Chapel Hill (hereafter UNCCH-186) [30], and a 159 patient cohort also with intrinsic subtype from University Hospital, Stockholm, Sweden (hereafter Stockholm-159) [31], the only significant results were Wnt (p = 0.001) and TGF-β (p = 0.035) in the Stockholm-159 basal-like BC subtype (Additional file 3: Figure S2D-F). Canonical pathway expression, assessed as the mean of normalized gene components, was measured for differential regulation between breast cancer subtypes. After correction for multiple hypotheses, the most notable pathways included Wnt signaling upregulated in the TNBC/basal-like BC subtypes of the Quebec, UNCCH-186, and Stockholm-159 cohorts; TGF-β in the TNBC subtypes of the Quebec and UNCCH-186 cohort; and both ErbB and VEGF signaling upregulated in the MSKCC-99 triple negative and UNCCH-186 basal-like subtypes (p < 0.05, Additional file 3: Figure S3). Of the 10 KEGG canonical signal transduction pathways investigated, Wnt was the most commonly overexpressed pathway in the TNBC subtypes indicating pathway perturbation in these types of breast cancers (Additional file 3: Figure S4). Based on these data we proceeded to examine experimental data sets to confirm these findings. Oncogenic signaling was investigated using gene sets derived from adenoviral vector transfected human mammary epithelial cells (HMECs) for β-catenin, E2F3, Myc, Ras, and Src genes [22]. Transcripts uniquely induced and inhibited from each HMEC model were used to define oncogenic pathway expression in the same manner as that applied to the canonical pathways, except in this case both those genes expressed and inhibited were used to measure pathway expression (see supplementary methods – HMEC Oncogenic Pathways). Analysis of pathway regulation by subtype indicated Wnt/β-catenin as the most commonly upregulated pathway in TNBC subtypes (Additional file 3: Figure S5). Indeed, the Wnt pathway was upregulated relative to the HR + or luminal subtypes in each cohort examined (Additional file 3: Figure S6). Thus, these data indicate that both oncogenic and canonical Wnt signaling pathways are uniquely upregulated in the TNBC subtypes. Expression of Wnt/β-catenin components in patients classified as Wnt-compared to Wnt+ (Wnt classifier signature) in each of the 11 studies analyzed in the meta-analysis shows greater expression of tumors classified as Wnt+ (Wnt classifier signature) (Additional file 3: Figure S7). To further validate Wnt transcriptional activation in TNBC subtypes an independent experimental Wnt gene set was used to analyze breast cancer subtypes. This Wnt classifier signature (mentioned in figure as Wnt+) is composed of 81 genes identified by treating lung cancer cell lines with the Wnt3A ligand (hereafter LWS-81) [24]. Despite a small overlap of the LWS-81 genes and probes available on the breast cancer DASL panel (n =15), this gene set clustered TNBC patients together in both Quebec and Georgia cohorts (Figure 1). Moreover, Wnt signaling defined by the LWS-81 genes was significantly upregulated in TNBC as compared HR + or luminal subtypes in the Quebec, Georgia, MSKCC-99, UNCCH-186, and Stockholm-159 studies (Figure 2). These data, in agreement with the β-catenin induced Wnt signaling from Bild et al. [22] (Additional file 3: Figure S5), uniformly indicate elevated oncogenic Wnt signaling in TNBC subtypes.

### Functional involvement of Wnt signaling in metastasis–associated tumor cell phenotypes

**Figure 3:**
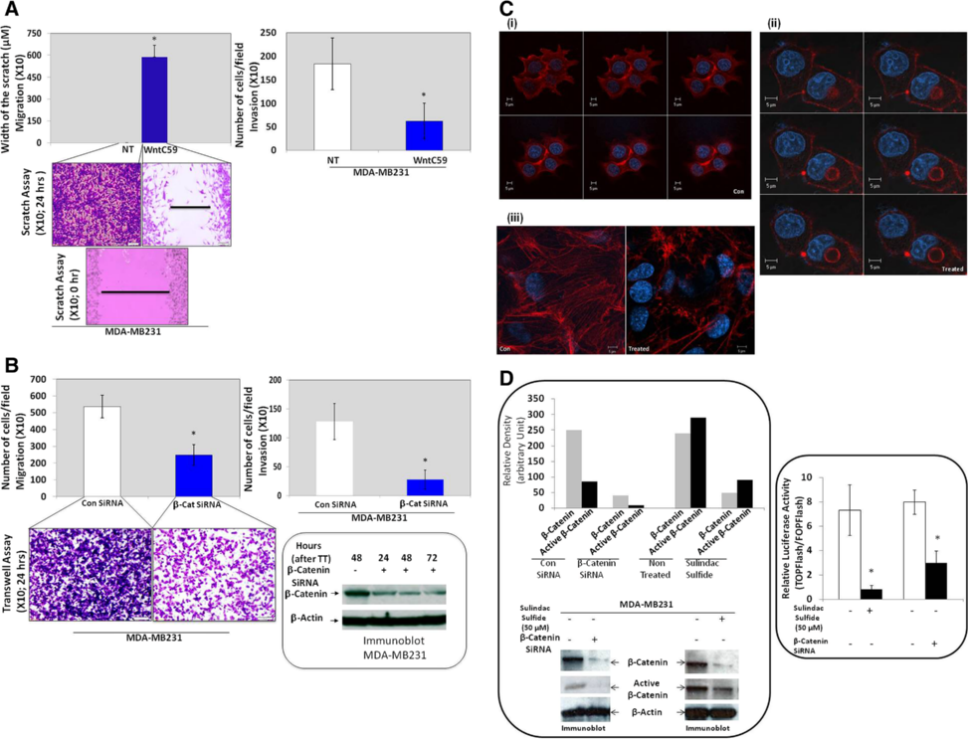
**Wnt/β-catenin pathway plays a critical role in the regulation of metastasis-associated phenotypes in TNBC cell line based models.** Functional blockade of Wnt/β-catenin pathway following the administration of Wnt-antagonist, WntC59 (10nM) **(A)**, and transient transfection of β-catenin SiRNA **(B)** caused a decrease in fibronectin-mediated migration (left panel), and invasion (right panel) in MDA-MB231 TNBC cells. Migration assays (scratch assay), and invasion assays were performed on WntC59 treated or β-catenin SiRNA transfected cells. Crystal violet stain was used for the semi-quantification (Olympus DP72; X10) **(A)**. Functional assays (transwell migration assay, and invasion assay) were carried out at 48 hours following transfection. Lysates from cells transiently transfected with scrambled SiRNA, and β-catenin SiRNA at 24, 48, and 72 hours were quantified for β-catenin expression by Western Blot (**B** Inset). Attenuator of Wnt-beta catenin signaling (sulindac sulfide) substantially abrogated the F-actin cytoskeletal organization in HCC38 TNBC cell line **(Cii)** as compared to the control **(Ci)** as well as in MDA-MB231 TNBC cell line **(Ciii)**. Active beta-catenin levels were semi-quantified in arbitrary units (Image J) following Western blot analyses from the clarified MDA-MB231 cell lysates (beta-catenin SiRNA transfected cells and sulindac sulfide treated cells. Upper bar diagrams showed the relative desitometric expressions of beta-catenin, and active beta-catenin. Beta-actin was used as the loading controls (**D** left panel). Relative luciferase activity (TOP Flash over FOP Flash) measured in MDA-MB231 cells following beta-catenin SiRNA transfection and sulindac sulfide treatment was plotted (three different experiments). Error bars represent standard error of the means (SDs), and statistical significance was determined by paired t-test. *P < 0.05 (**D** right panel).

TNBC is a highly metastatic disease. A transcriptionally active β-catenin (unphosphorylated on Serines 33 and 37 as well as Threonine 41) [36] is a direct functional readout of Wnt/β-catenin signaling. As an independent line of evidence for Wnt pathway activation in TNBC, and its functional association with the metastatic disease, the role of Wnt signaling was examined *in vitro* using TNBC cell lines. A functional blockade of Wnt/β-catenin pathway by either a pharmacological Wnt-antagonist, WntC59, or β-catenin SiRNA mediated genetic manipulation demonstrated that a functional perturbation of the pathway is causal to the metastasis- associated phenotypes including fibronectin-mediated and invasion in TNBC cells. Similarly, Wnt/β-catenin signaling attenuator, sulindac sulfide also inhibited migration of TNBC cells on fibronectin (data not shown). We observed that the treatment with a pharmacological Wnt-antagonist, WntC59 blocked fibronectin-mediated migration and invasion in MDA-MB231 cells (Figure 3A). This is consistent with our observation that the SiRNA-mediated decrease in levels of β-catenin protein at different time points following tranfection of β-catenin SiRNA in MDA-MB231 cells (Figure 3B) caused significant inhibition of fibronectin-mediated migration and invasion. Since F-actin organization is one of the key effectors of cell movement, we tested the effect of inhibition of Wnt-signals on the F-actin organization in TNBC cells. Out data show that sulindac sulfide substantially abrogated the cellular organization of F-actin (Z-section) on fibronectin in MDA-MB231 and HCC38 TNBC cells (Figure 3C i, ii and iii). Finally, we carried out a real time video microscopy of the cell movement following the inhibition of Wnt-signals in HCC38 TNBC cells (Additional files 5 and 6; two AVI files).

To address the issue of whether or not the modulation of Wnt-beta-catenin pathway causes the attenuation of its downstream signals, we tested the expression of active beta-catenin following beta-catenin SiRNA or sulindac sulfide treatment in MDA-MB-231 cells (Figure 3D left panel). Since active beta-catenin levels were decreased in both the conditions, we also conducted the experiment to study the transcriptional activity of beta-catenin. Our data show a significant decrease in the relative luciferase activity following beta-catenin SiRNA or sulindac sulfide treatment in MDA-MB-231 cells (Figure 3D right panel).

### Wnt/β-catenin signaling is associated with metastatic disease

**Figure 4:**
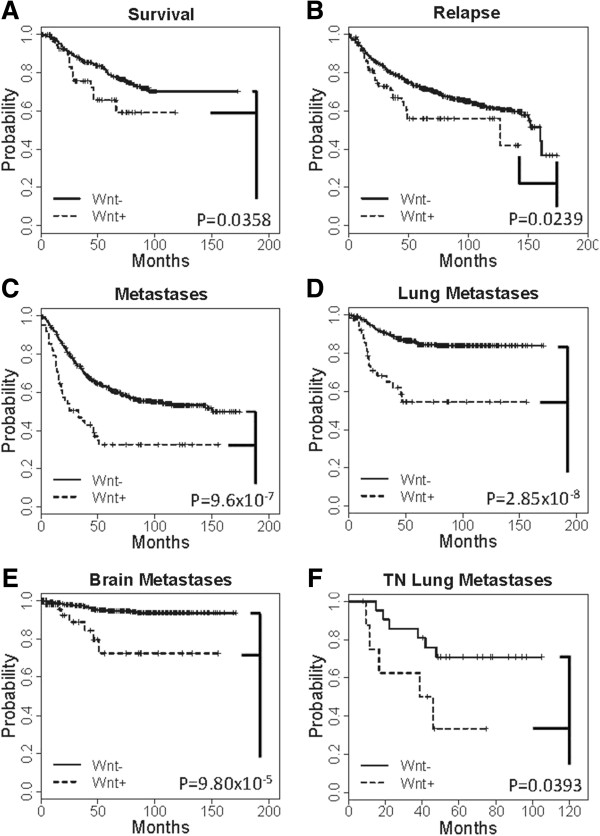
**Kaplan-Meier survival curves of Wnt/β-catenin positive (Wnt+: Wnt classifier signature) and Wnt/β- catenin negative (Wnt-) patients with respect to A) overall survival, B) recurrence-free survival, C) metastasis-free survival, D) lung metastasis-free survival, E) brain metastasis-free survival, and F) lung metastasis-free survival in TNBC patients.** P-values were calculated by the log-rank test.

**Figure 5:**
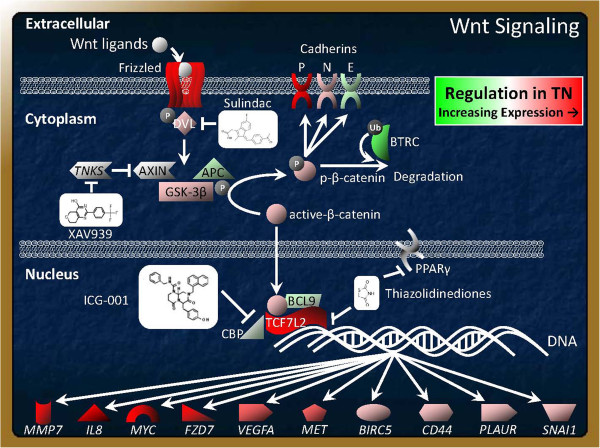
**Wnt signaling schematic identifying well known mechanisms of interaction overlaid with gene expression regulation in TNBC tumors in the Quebec, Georgia, and MSKCC-99 cohorts and basal-like carcinomas in the UNCCH-186 and Stockholm-159 cohorts.** Upregulated components include frizzled receptors, which when bound by Wnt ligands sequester disheveled (DVL1), breaking up a β-catenin phosphorylating complex composed of Axin, APC, and GSK-3β. This complex, when intact phosphorylates β- catenin on Serines 33, 37, and Threonine 41. Phosphorylated β-catenin can either become poly-ubiquitinated by the phosphate dependent ubiquitin ligase, BTRC, and subsequently degraded by the proteasome, or phosphorylated β-catenin can bind to P, N, and E type cadherins that enhance cytosolic β-catenin turnover. When the DVL, Axin, GSK-3β, and APC β-catenin phosporylating complex is not intact β-catenin does not become phosphorylated leading to transcriptional active β-catenin which can translocate to the nucleus and transcribes Wnt transcriptional target in combination with other co-activators including TCF7L2 (also known as TCF4), BCL9, and CREB binding protein (CBP).

**Figure 6:**
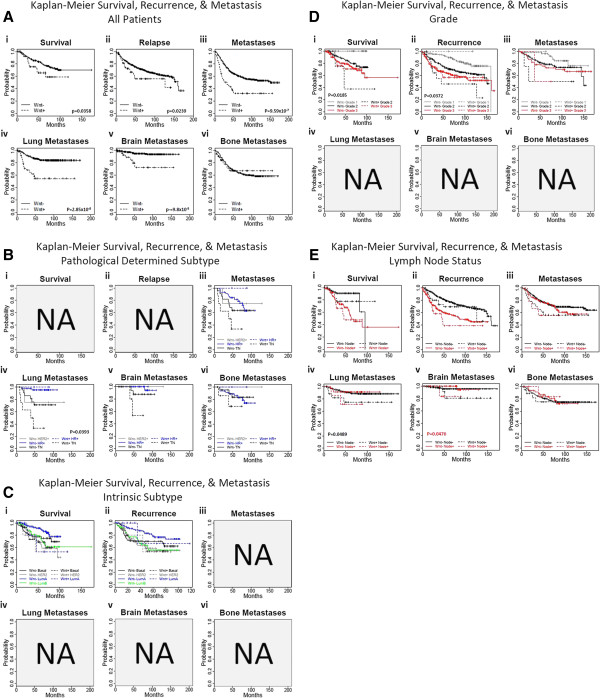
**Kaplan-Meier survival analyses were performed on the data.** Kaplan-Meier survival analyses for i) overall, ii) recurrence-free, iii) metastasis-free,Metastasis-free, iv) lung metastasis-free, v) brain metastasis-free, and vi) bone metastasis-free survival for patients classified as Wnt + (Wnt classifier signature) and Wnt- for **A)** all patients and patients stratified by **B)** pathological determined subtype, **C)** intrinsic subtype, **D)** histological grade, and **E)** lymph node status. Only significant (p < 0.05) p-values are shown and are color-coded to correspond to the respective stratum.

We have performed Kaplan-Meier survival curves of Wnt/β-catenin positive (Wnt+: Wnt classifier signature) and Wnt/β- catenin negative (Wnt-) patients with respect to overall survival, recurrence-free survival, metastasis-free survival, lung metastasis-free survival, brain metastasis-free survival, and lung metastasis-free survival in TNBC patients (Figure 4). To investigate the implications of Wnt signaling, we used a nearest shrunken centroid classifier [33] to stratify patients by Wnt/β-catenin transcriptional activity. This Wnt/β-catenin classifier was trained to identify β-catenin transformed HMECs as opposed to normal and other oncogenic (E2F3, Myc, Ras, and Src) models [22] and subsequently applied to a meta-analysis of 11 studies and 1,878 expression profiles from primary breast cancers [25-32,37] including the Quebec and Georgia cohorts. Well known components of the Wnt signaling pathway is represented in Figure 5. Wnt/β-catenin positive (Wnt+) tumors accounted for 188 of the 1,878 patients. Patients were subsequently analyzed for intrinsic and pathology determined subtype, lymph node status, and grade, as well as metastasis-free (MFS), recurrence-free (RFS), and overall survival (OS). Complete pathology records for ER, PR, and HER2 were available for 310 patients, and of these 56 were categorized as Wnt+, 52 of which were TNBC. This strongly supports earlier observations that Wnt/β-catenin is preferentially activated in the TNBC subtype (p = 6.3 × 10-14, Fisher’s exact test). Analysis of 465 patients with intrinsic subtype found 53 of 71 Wnt + tumors corresponded to the basal-like subtype, likewise implicating elevated Wnt signaling in this subtype (p = 2.2 × 10-16). Data regarding spread of disease to lymph nodes were available for 1,202 patients where Wnt + patients composed 7.1% and 10.2% of lymph node negative and positive cases, respectively, marginally associating Wnt signaling with positive lymph node status (p = 0.042). In contrast, analysis of 912 patients with histological grades 1, 2, and 3 consisted of 1.1%, 5.7%, and 15.2% of Wnt + patients, correlating Wnt signaling with grade 3 carcinomas (p = 4.0 × 10-11, Additional file 3: Table S3). These data support our earlier observations of elevated Wnt signaling in TNBC subtypes, and associate Wnt signaling with high grade carcinomas. Survival analyses of breast cancer patients with elevated Wnt signaling distinguish these cancers as having greater metastatic potential and overall worse prognoses. Kaplan-Meier survival curves for OS, RFS, and MFS, including lung, brain, and bone specific metastases where analyzed for Wnt + and Wnt- patients stratified by pathology and intrinsic subtypes, grade, and lymph node status (Figure 6). Significantly increased risk was found for Wnt + patients with respect to OS, RFS, and MFS (p < 0.05, Figure 4). However, these differences were most significant in the metastatic setting (p = 9.6 × 10-7) and specifically in lung and brain but not bone metastases (Figure 6A vi). Stratifying patients by pathological determined subtype limited this analysis to a much smaller cohort of patients with both outcome and pathology records; however, Wnt + TNBC patients had greater risk of lung metastases (p = 0.0393, Figure 4F). Other significant differences include a worse prognosis for Grade 2 Wnt + patients with respect to OS as well as RFS (Figure 6D), and increased risk for lung and brain metastases for lymph node negative and positive patients, respectively (Figure 6E). These data cumulatively suggest that increased Wnt/β-catenin signaling is associated with metastatic pathways to the brain and lung.

## Discussion

Our study demonstrates two major findings that are consistent with current knowledge and advance our understanding of subset specific breast cancer etiology. First, using novel technologies to characterize FFPE materials we ascertained data consistent with clinical pathological subtype that also identified canonical and oncogenic Wnt signaling as an activated pathway in TNBC. These findings were confirmed using multiple published studies in diverse cohorts of patients across different microarray platforms, as well as independent experiments that identified Wnt induced targets which were consistently upregulated in TNBC subtypes. In the context of reports indicating associations of Wnt signaling with the basal-like subtype [38,39], these data strongly suggest that the Wnt pathway is preferentially activated in TNBC subtypes, and may represent a possible therapeutic target in the treatment of these cancers. Reis-Filho’s group reported that β-catenin pathway activation in BC is associated with the TNBC phenotype but not with CTNNB1 mutation [12]. We have observed (by immuno-fluorescence) a higher level of β-catenin (active) in the nucleus of MDA-MB231 TNBC cells as compared to non-TNBC (MCF7 and BT474) cell lines (data not shown).

Khramtsov et al., and others reported the association of Wnt signaling in TNBC with higher metastasis and poor prognosis [40,41]. This observation can be explained by the fact that Wnt-β-catenin pathway plays a critical role in the regulation of metastasis-associated phenotypes in tumor cells. Breast cancer metastases are osteolytic in nature, and osteolytic bone lesions are formed due to tumor-induced bone resorption and destruction [42]. Regulatory mechanisms underlying osteolytic metastasis to bone is a vicious cycle reflecting complex interplay of molecules which is propagated by four contributors: tumor cells, osteoblasts, osteoclasts and factors within bone matrix [43,44]. Wnt-pathway has emerged as a crucial regulator of bone formation, and regeneration as Wnt signaling stimulates bone formation, and is also reported as a therapeutic target for bone diseases [45,46]*.* Wnt signaling in osteoblasts regulates expression of the receptor activator of NFkappaB ligand, and inhibits osteoclastogenesis *in vitro*[47] (while Dickkopf1, a secreted Wnt/beta-catenin antagonist, produced by breast tumor cells is an important mechanistic link between primary breast tumors, and secondary osteolytic bone metastases [42]. In case of hormone-receptor positive tumors, it has been reported that in contrast to its role in breast cancer initiation, estrogen signaling has a protective effect in later stages, where estrogen receptor (ER) loss associates with aggressive metastatic disease [48]. In the context of the above mentioned reports, it appears that Wnt-positivity in hormone-receptor positive patients (HR+/Wnt+) may have a negative regulatory influence on bone metastases as observed by the lower rate of bone metastatic events as compared to hormone receptors-positive Wnt-negative patients (HR+/Wnt-).

In our data, both TNBC and HER2+ have somewhat similar/identical average levels of wnt-expression, and expression of wnt/beta-catenin genes (Figure 2 and Additional file 3: Figure S5). This similarity in the expression pattern(s) between the TNBC and the HER2+ group can be due to an intrinsic heterogeneity within HER2-enriched/amplified subtype. Indeed, it has been shown that, when only HER2-amplified breast cancers are taken into account, approximately 50% are ER-positive [49]. Hence the other 50% are ER-negative HER2-amplified breast cancers. Previous comparative genomic hybridization (CGH) studies have demonstrated that ER- negative disease differs significantly from ER-positive cancers in terms of the pattern, type, and complexity of genetic aberrations [50-53]. Furthermore, data at the European Society for Medical Oncology (ESMO) Vienna 2012 congress on the duration of adjuvant trastuzumab therapy hint at a difference between HER2 + /ER + and HER2 + /ER − disease, in keeping with the concept that HER2 + /luminal is biologically distinct from HER2 + /HER2-enriched disease, which is predominantly ER − [30,54]*.* Sircoulomb et al. have shown that ER + and ER- ERBB2-amplified BCs are different and the WNT/b catenin signaling pathway was involved in ERERBB2-amplified BCs [55]. Thus it is highly possible that ER-negative HER2-amplified tumors present within the HER2-amplified group in our study can influence the levels of wnt-expression and expression of wnt/β-catenin genes.

Wnt-C59 is a potent and selective Wnt signaling modulator with IC50 <0.11 nM in Wnt-Luc reporter assay for Wnt pathway inhibition, and with chemical/physical properties, suitable for *in vitro*/*in vivo* studies. Wnt-C59 prevents palmitylation of Wnt proteins by Porcupine, thereby blocking Wnt secretion and activity, similar to Wnt inhibitors IWP-2, IWP-3 and IWP-4. The observed inhibition of integrin-directed migration and invasion of MDA-MB231 cells following Wnt-C59 treatment in our results provides mechanistic explanation to our observation that, (1) Wnt signaling is upregulated in TNBC, and (2) Wnt/β-catenin signaling is associated with metastatic disease. Recently, Craig et al., have reported genome and transcriptome sequencing in prospective metastatic triple negative breast cancer [56]. To further ascertain the functional significance of the pathway in metastatic disease, we also genetically manipulated the cellular levels of β-catenin, the functional readout of Wnt/β-catenin pathway (Figure 5). Together, our functional data demonstrate a direct involvement of Wnt-β-catenin pathway in the metastasis-associated phenotypes in tumor cell (Figure 3 and Additional file 5). We have studied the cause-effect relationship of Wnt-β-catenin pathway with metastasis in TNBC cell line models using three tools, genetic (beta-catenin SiRNA), pharmacological (Wnt-β-catenin pathway modulator; Wnt-C59), and functional (sulindac sulfide). Our data show that perturbation of the Wnt-β-catenin pathway abrogated metastasis-associated phenotypes in TNBC cells following attenuation of beta-catenin transcriptional activity, proving a direct mechanism based relationship between Wnt-β-catenin pathway and metastasis in TNBC. The results of the study may have implications for therapeutic target identification in future. The functional data would benefit from validation in other *in vitro* models.

Furthermore, other observations in this analysis include upregulation of Myc regulated genes (Additional file 3: Figure S5) that is consistent with recent reports identifying this Wnt transcriptional target [57] as upregulated in the basal-like subtype [58]. Thus, these data indicate that a significant subset of TNBC is characterized by Wnt activation. Notable Wnt transcriptional targets upregulated in TNBC (Figure 5) included matrix metallopeptidase 7 (MMP7) [59], inteleukin 8 (IL8) [60], MYC [57], VEGF [61], frizzled 7 (FZD7) [62], survivin (BIRC5) [61], CD44 [63], MET [38,64], peroxisome proliferator-activated receptor gamma (*PPARD*) [65], uPAR (PLAUR) [66], and snail (SNAI1) [67] (Figure 5). Furthermore, several Wnt antagonists were downregulated in the TNBC subtypes such as the androgen receptor (AR) [68,69], FOXA1 [70], and MYB [71]. These data highlight some of the Wnt components differentially regulated in TNBC.

In addition to the observed upregulation of Wnt signaling in TNBC, we also found association of Wnt signaling with metastatic disease. The Wnt/β-catenin classifier trained to identify oncogenic β-catenin signaling identified a disproportionate number of TNBC patients, supporting earlier observations of preferential Wnt activation in this subtype. Importantly, this classifier identified patients that were more likely to experience lung and brain metastases. These two metastatic routes have been associated with the basal-like subtype where Wnt signaling was noted as upregulated [39], and more recently, Wnt has been causally implicated in lung metastases [24]. Our analyses further establish these findings and suggest that Wnt signaling confers a greater risk of lung metastases within the TNBC subtype (Figure 4F). There are certain limitations of the study. Sample size of HER2 enriched/amplified group in both Quebec and Georgia cohorts is lower than HR + and TNBC groups. We have restricted our study only to the 3 major clinical treatment categories of breast cancer, HR+, HER2+ and TNBC based on comprehensive gene expression profiling. However, clinically, each of three major categories of breast cancer is also a heterogeneous group by themselves. Several recent studies have described that even the relatively small class of breast tumors like TNBC can be further divided into five or six subclasses, each with its own molecular features, and unique sensitivity to therapeutic agents. A number of hypotheses have been proposed to explain the origin of inter-tumor heterogeneity in breast cancer, including subtype-specific tumor cell–of–origin and transforming events [72,73]. We also have not stratified our study in the node-negative and node-positive settings.

Targeting the Wnt pathway has traditionally been difficult, but emerging modalities provide potential opportunities. Examples of small molecule inhibitors include sulindac, XAV939, ICG-001, and thiazolidinediones (TZDs) (Figure 5). Sulindac is a non-steroidal anti-inflammatory drug (NSAID) which also inhibits Wnt signaling by binding the PDZ domain of disheveled (DVL1) [74,75] and like other NSAIDS, inhibits cyclooxygenase-2 (COX2), a gene recently implicated in breast cancer metastasis to the brain [25,76]. XAV939 is a small molecule inhibitor that has recently been identified as targeting the poly-ADP ribose polymerase (PARP) gene tankyrase, which degrades Axin and allows β-catenin to avoid phospohorylation, subsequent poly-ubiquitination, and proteasomal degradation. We have observed that both sulindac and XAV939 blocks metastasis associated phenotypes (e.g. integrin-dependent migration, matrigel invasion, vascular mimicry) and clonogenic growth in TNBC cells lines (data not shown).

## Conclusion

Our study contributes not only by identifying clinical markers associated with Wnt signaling such as histological grade 3 tumors and TNBC pathological subtype, but also increased risk for brain and lung metastases, thus recognizing Wnt signaling as a rational target in TNBC. The results of the study have implications for therapeutic target identification and the design of future clinical trials for this aggressive group of breast cancer. More genomic studies like this, however, are needed to create a genetic landscape of TNBC which will be utilized to differentiate “driver mutations” from “carrier mutations” and will guide therapeutics development. Individualized treatment will be possible only once we fully appreciate the biology of these genetic abnormalities.

## Additional files

**Introduction to Additional file 3:** Details of Figure S1: Assay reproducibility in the Quebec cohort measured by a distribution of Pearson R2 coefficients between RNA replicates on the A) human, and B) breast cancer (BC) DASL panels. C) Overlap of gene content on the human (dashed line) and BC (solid line) DASL panels, and D) replicated samples on both platforms. E) Distribution of inter-platform Pearson R2 correlations for samples run on both platforms, and F) a Venn diagram of differentially regulated genes found using the overlap of patients and genes on the human and BC DASL panels. Expression of ESR1, PGR, and ERBB2 by pathology determined subtype for HR + (white), HER2+ (grey), and TN (black) subtypes in the Quebec cohort on the G) human and I) BC DASL panels, as well as the K) Georgia cohort on the BC DASL panel correspond with expected clinical pathological subtype. Cohort sizes by pathology subtype for the Quebec cohort on the H) human and J) BC DASL panels, as well as the L) Georgia cohort on the BC DASL panel are depicted by pie charts. Details of Figure S2: Analysis of upregulated probes in TNBC as compared to other subtypes in context with KEGG [21] signal transduction pathways. Overrepresented probes in each pathway were compared to the number of pathway probes available on the specific platform and the total number of probes upregulated using Fisher’s exact test. A) Quebec cohort on the human cancer DASL panel (QC-HCP), B) Quebec on the BC DASL panel (QC-BCP), C) Georgia cohort on the BC DASL panel (GA-BCP), D) MSKCC-99 [29], E) UNCCH-186 [30], and F) Stockholm-159. P-values were Bonferroni corrected and significant pathways (p <0.05) are in bold. Details of Figure S3: Pathway expression of canonical KEGG signal transduction pathways [21] were measured as the normalized mean of the pathway components and subsequently used to calculate pathway perturbation between BC subtypes (see Additional file 4). Pathways that were differentially expressed relative to the TNBC subtype have significance lines and a corresponding p-value with Bonferroni’s correction for multiple hypothesis testing applied. Analyses included cohorts from Quebec profiled on the A) human (QCHCP) and B) BC DASL panels (QC-BCP), C) Georgia on the BC DASL panel (GA-BCP), D) MSKCC-99 [29], E) UNCCH-186 [30] and F) Stockholm-159 [31]. Details of Figure S4: Canonical Wnt expression of KEGG [21] pathway components. Pathway regulation was higher in TNBC as compared to other subtypes in A & B) Quebec (QC-HCP & QC-BCP), C) Georgia (GABCP), D) MSKCC-99 [29], E) UNCCH-186 [30], and F) Stockholm-159 cohorts. Significant p-values after Bonferroni’s correction are shown relative to the TNBC subtypes (see Additional file 4). Details of Figure S5: Pathway expression of experimentally derived oncogenic signaling pathways from Bild et al. [22] measured between BC subtypes (see Additional file 4). Pathways that were differentially expressed after Bonferroni’s correction are in bold. Analysis include cohorts from Quebec profiled on the A) human (QC-HCP) and B) BC DASL panels (QCBCP), C) Georgia on the BC DASL panel (GA-BCP), D) MSKCC-99 [29], E) UNCCH-186 [30], and F) Stockholm-159 [31]. Hierarchical clustering of pathway expression depicts patterns in pathway regulation (rows) in context with BC subtype (columns). Details of Figure S6: Experimentally induced Wnt/β-catenin pathway expression from Bild et al. [22]. Pathway regulation was assessed in the Quebec cohort on the A) human (QC-HCP) and B) BC DASL panels (QC-BCP), C) Georgia (GA-BCP), D) MSKCC-99 [29], E) UNCCH-186 [30], and F) Stockholm-159 cohorts. Significant p-values after Bonferroni’s correction are shown relative to the TNBC subtypes.

## Abbreviations

TN: Triple negative; HR: Hormone receptor; ER: Estrogen receptor; PR: Progesterone receptor; HER2: Human epidermal growth factor receptor 2; OS: Overall survival; RFS: Recurrence-free survival; MFS: Metastasis-free survival; DASL: cDNA mediated annealing, selection, and ligation.

## Competing interests

The authors declare that they have no competing interests.

## Authors’ contributions

ND was involved in designing / conducting the cell-line based experiments, and actively contributed to writing the manuscript. BB is the key contributor in the data analyses, and one of the key authors on writing the manuscript together with ND. CM helped design the DASL component of the study, performed the DASL analysis, and contributed to writing the manuscript. MO prepared RNA. ZC performed statistical analysis. PD contributed in designing experiments and contributed to writing the manuscript. WT prepared RNA and performed some DASL experiments. CC conducted pathological analysis. KK was involved in coordinating the study. GS conceived of the study, and participated in its design. MA participated in the design of the study and helped to draft the manuscript. MB helped conceive and design the molecular component of the study and contributed to writing the manuscript. BLJ participated in the overall conception and the design of the study. All authors read and approved the final manuscript.

## Pre-publication history

The pre-publication history for this paper can be accessed here:

http://www.biomedcentral.com/1471-2407/13/537/prepub

## Supplementary Material

Additional file 1: Table S1 Illumia Cancer Panel. Click here for file

Additional file 2: Table S2 Custom Cancer Panel. Click here for file

Additional file 3 **Figure S1-Table S3:** Assay reproducibility in the Quebec cohort measured by a distribution of Pearson R2 coefficients between RNA replicates. **Figure S2:** Analysis of upregulated probes in TNBC as compared to other subtypes in context with KEGG [21] signal transduction pathways. P-values were Bonferroni corrected and significant pathways (p <0.05) are in bold. **Figure S3:** Pathway expression of canonical KEGG signal transduction pathways [21] were measured as the normalized mean of the pathway components and subsequently used to calculate pathway perturbation between BC subtypes (see Additional file 4). **Figure S4:** Canonical Wnt expression of KEGG [21] pathway components. Significant p-values after Bonferroni’s correction are shown relative to the TNBC subtypes (see Additional file 4). **Figure S5:** Pathway expression of experimentally derived oncogenic signaling pathways from Bild et al. [22] measured between BC subtypes (see Additional file 4). Hierarchical clustering of pathway expression depicts patterns in pathway regulation (rows) in context with BC subtype (columns). **Figure S6:** Experimentally induced Wnt/β-catenin pathway expression from Bild et al. [22]. Significant p-values after Bonferroni’s correction are shown relative to the TNBC subtypes. **Figure S7:** Expression of Wnt/β-catenin components in patients classified as Wnt compared to Wnt + (Wnt classifier signature) in each of the 11 studies analyzed in the meta-analysis shows greater expression of tumors classified as Wnt + (Wnt classifier signature). **Table S3:** Table identifying the number of patients from each cohort in a meta-analysis of 11 studies and 1,878 patients with pathological or intrinsic determined subtype, grade, and lymph node status. Each category is broken down by total number of Wnt + (Wnt classifier signature) and Wnt- patients identified by the Wnt/β-catenin classifier and then analyzed for overrepresentation using Fisher’s exact test. Click here for file

Additional file 4 Supplementary Analysis and Statistical Methods. Click here for file

Additional file 5 **Real-time Video Microscopy: A scratch-would healing assay was performed on the confluent layer of cells (grown on fibronectin-coated glass-cover slip culture- dishes; Mattek, Ashland, MA). **Time-lapse images are acquired with a Perkin Elmer Ultraview ERS (Norwalk, CT) disk-spinning confocal system, mounted on a Zeiss Axiovert 200 M inverted microscope equipped with a 37°C stage warmer, incubator, and humidified CO2 perfusion system. Bright-field images are acquired with a Hamamatsu Orca-ER camera with a Plan-Neoflour 10x objective (NA 0.75; 1x1 binning) at 10 minutes intervals for each image set. HCC38 cells were treated with sulindac sulfide, and their movement was compared with the vehicle treated cells. Click here for file

Additional file 6 Real-time Video Microscopy. Click here for file
