# Supplementary material for: Wnt signaling in triple negative breast cancer is associated with metastasis
Source: BMC Cancer. 2013 Nov 10;13:537. doi: 10.1186/1471-2407-13-537 (PMC4226307; doi:10.1186/1471-2407-13-537)
Supplement: Additional file 1: Table S1 — Illumia Cancer Panel. [file 1471-2407-13-537-S1.pdf]

Supplementary Table 2

Gene Symbol	Gene identifiers	Alias Symbols	Chromosomal Location
ABCB1	5243	ABC20;CD243;GP170;MDR1;P-gp;PGY1	7q21.1
ABCC2	1244	ABC30;CMOAT;DJS;MRP2;cMRP	10q24
ABCG2	9429	ABC15;ABCP;BCRP;BCRP1;BMDP;EST157481;MRX;MXR;MXR1	4q22
ABL1	25	ABL;JTK7;c-ABL;p150;v-abl	9q34.1
ADPRT	142	ADPRT1;PARP;PARP-1;PPOL;pADPRT-1	1q41-q42
AHR	196		7p15
AIM2	9447		1q22
AKT1	207	PKB;PRKBA;RAC;RAC-ALPHA	14q32.32
AKT2	208	PKBBETA;PRKBB;RAC-BETA	19q13.1-q13.2
ALK	238		2p23
ALOX12	239	LOG12	17p13.1
APAF1	317	CED4	12q23
APC	324	DP2;DP2.5;DP3;FAP;FPC;GS	5q21-q22
AR	367	AIS;DHTR;HUMARA;KD;NR3C4;SBMA;SMAX1;TFM	Xq11.2-q12
ARAF1	369	A-RAF;PKS2;RAFA1	Xp11.4-p11.2
AREG	374	AR;CRDGF;MGC13647;SDGF	4q13-q21
ARHA	387	ARH12;RHO12;RHOA;RHOH12	3p21.3
ARHGDIB	397	D4;GDIA2;GDID4;LYGDI;RAP1GN1	12p12.3
ARHH	399	RHOH;TTF	4p13
ARHI	9077	NOEY2	1p31
ARNT	405	HIF-1beta;HIF1B;HIF1BETA;HIF1beta;TANGO	1q21
ATF1	466	TREB36	12q13
ATM	472	AT1;ATA;ATC;ATD;ATDC;ATE	11q22-q23
AXL	558	UFO	19q13.1
BAD	572	BBC2;BCL2L8	11q13.1
BAG1	573		9p12
BAK1	578	BAK;BCL2L7;CDN1	6p21.3
BARD1	580		2q34-q35
BCL2	596	Bcl-2	18q21.3
BCL2A1	597	BCL2L5;BFL1;GRS;HBPA1	15q24.3

BCL2L1	598	BCL-XL/S;BCL2L;BCLX;Bcl-X;bcl-xL;bcl-xS	20q11.21
BCL3	602	BCL4	19q13.1-q13.2
BCL6	604	BCL5;LAZ3;ZNF51	3q27
BCR	613	ALL;BCR1;CML;D22S11;D22S662;PHL	22q11.23
BIRC2	329	API1;CIAP1;HIAP2;MIHB;RNF48	11q22
BIRC3	330	AIP1;API2;CIAP2;HAIP1;HIAP1;MIHC;RNF49	11q22
BIRC5	332	API4;EPR-1;SURVIVIN	17q25
BLM	641	BS;RECQ2;RECQL2;RECQL3	15q26.1
BMI1	648	MGC12685;RNF51	10p11.23
BMP4	652	BMP2B;BMP2B1;ZYME	14q22-q23
BRAF	673	BRAF1;RAFB1	7q34
BRCA1	672	PSCP;RNF53	17q21
BRCA2	675	FAD;FAD1;FANCB;FANCD1	13q12.3
BTK	695	AGMX1;AT;ATK;BPK;IMD1;PSCTK1;XLA	Xq21.33-q22
CASP10	843	ALPS2;FLICE2;MCH4	2q33-q34
CASP2	835	CASP-2;ICH-1L;ICH-1L/1S;ICH1;NEDD2	7q34-q35
CASP3	836	APOPAIN;CPP32;CPP32B;SCA-1	4q34
CASP8	841	FLICE;MACH;MCH5	2q33-q34
CAV1	857	CAV;VIP21	7q31.1
CBFA2T1	862	AML1T1;CDR;ETO;MGC2796;MTG8;ZMYND2	8q22
CBL	867	C-CBL;CBL2;RNF55	11q23.3
CBLB	868	RNF56	3q13.11-q13.12
CCNA2	890	CCN1;CCNA	4q25-q31
CCNC	892		6q21
CCND1	595	BCL1;D11S287E;PRAD1;U21B31	11q13
CCND2	894	KIAK0002	12p13
CCND3	896		6p21
CCNE1	898	CCNE	19q12
CCNH	902	CAK;p34;p37	5q13.3-q14
CD34	947		1q32
CD44	960	CD44R;IN;MC56;MDU2;MDU3;MIC4;Pgp1	11p13
CD59	966	MIC11;MIN1;MIN2;MIN3;MSK21;PROTECTIN	11p13
CD9	928	BA2;DRAP-27;MIC3;MRP-1;P24	12p13.3
CDC2	983	CDK1	10q21.1
CDC25A	993		3p21
CDC25B	994		20p13
CDC25C	995	CDC25	5q31

CDH1	999	CDHE;ECAD;LCAM;UVO	16q22.1
CDH11	1009	CAD11;CDHOB;OB;OSF-4	16q22.1
CDH13	1012	CDHH	16q24.2-q24.3
CDK10	8558	PISSLRE	16q24
CDK2	1017	p33(CDK2)	12q13
CDK4	1019	CMM3;MGC14458;PSK-J3	12q14
CDK6	1021	PLSTIRE	7q21-q22
CDK7	1022	CAK1;CDKN7;STK1;p39MO15	5q12.1
CDK9	1025	C-2k;CDC2L4;PITALRE;TAK	9q34.1
CDKN1A	1026	CAP20;CDKN1;CIP1;MDA-6;P21;SDI1;WAF1	6p21.2
CDKN1B	1027	CDKN4;KIP1;P27KIP1	12p13.1-p12
CDKN2A	1029	ARF;CDK4I;CDKN2;CMM2;INK4;INK4a;MLM;MTS1;P16;TP16;p14;p14ARF;p16;p16INK4;p19	9p21
CDKN2B	1030	INK4B;MTS2;P15;TP15	9p21
CDKN2C	1031	INK4C;p18;p18-INK4C	1p32
CDKN2D	1032	INK4D;p19;p19-INK4D	19p13
CEACAM1	634	BGP;BGP1;BGPI;CD66;CD66A;CD66a	19q13.2
CEBPA	1050	C/EBP-alpha;CEBP	19q13.1
CHEK1	1111	CHK1	11q24-q24
COL18A1	80781	KNO	21q22.3
COL1A1	1277	OI4	17q21.3-q22.1
COL4A3	1285	TUMSTATIN	2q36-q37
COMT	1312		22q11.21
COPEB	1316	BCD1;CPBP;GBF;KLF6;PAC1;ST12;ZF9	10p15
CREBBP	1387	CBP;RSTS;RTS	16p13.3
CRK	1398	CRKII	17p13.3
CRKL	1399		22q11.21
CSF1R	1436	C-FMS;CD115;CSFR;FIM2;FMS	5q33-q35
CSF2	1437	GMCSF	5q31.1
CSF3	1440	G-CSF;GCSF;MGC45931	17q11.2-q12
CSF3R	1441	CD114;GCSFR	1p35-p34.3
CSK	1445		15q23-q25
CSPG2	1462	VERSICAN	5q14.3
CTGF	1490	CCN2;IGFBP8;NOV2	6q23.1
CTNNA1	1495	CAP102	5q31
CTNNB1	1499	CTNNB	3p21
CTSD	1509	CPSD;MGC2311	11p15.5
CTSL	1514	CATL;MEP	9q21-q22

CUL2	8453		10p11.22
CXCL9	4283	CMK;Humig;MIG;SCYB9;crg-10	4q21
CYP1A1	1543	AHH;AHRR;CP11;CYP1;P1-450;P450-C;P450DX	15q22-q24
CYP1B1	1545	CP1B;GLC3A	2p21
DAB2	1601	DOC-2;DOC2	5p13
DAP3	7818	DAP-3;MRP-S29;MRPS29;bMRP-10	1q21-q22
DAPK1	1612	DAPK	9q34.1
DCC	1630	CRC18;CRCR1	18q21.3
DCN	1634	DSPG2;PG40;PGII;PGS2;SLRR1B	12q13.2
DDB2	1643		11p12-p11
DDIT3	1649	CEBPZ;CHOP;CHOP10;GADD153	12q13.1-q13.2
DDX6	1656	HLR2;P54;RCK	11q23.3
DEK	7913	D6S231E	6p23
DKC1	1736	DKC;NAP57;NOLA4;XAP101;dyskerin	Xq28
DLC1	10395	ARHGAP7;FLJ21120;HP;STARD12;p122-RhoGAP	8p22
DLEU1	10301	BCMS;LEU1;XTP6	13q14.3
DLG3	1741	KIAA1232;NEDLG;SAP102	Xq13.1
DMBT1	1755	GP340	10q25.3-q26.1
DSP	1832	DPI;DPII;KPPS2;PPKS2	6p24
DTR	1839	DTS;HBEGF;HEGFL	5q23
DVL3	1857	KIAA0208	3q27
E2F1	1869	E2F-1;RBBP3;RBP3	20q11.2
E2F2	1870	E2F-2	1p36
E2F3	1871	E2F-3;KIAA0075	6p22
E2F5	1875	E2F-5	8q21.2
EGF	1950	URG	4q25
EGFR	1956	ERBB;ERBB1	7p12
EGR1	1958	AT225;KROX-24;NGFI-A;TIS8;ZIF-268;ZNF225	5q31.1
ELK1	2002		Xp11.2
ELK3	2004	ERP;NET;SAP2	12q23
ELL	8178	ELL1;Men	19p13.1
EMS1	2017	Cttn	11q13
ENC1	8507	CCL28;ENC-1;NRPB;PIG10;TP53I10	5q12-q13.3
EPHA1	2041	EPH;EPHT;EPHT1	7q34
EPHB4	2050	HTK;MYK1;TYRO11	7q22
EPO	2056	EP	7q22
EPS15	2060	AF-1P;AF1P;MLLT5	1p32
EPS8	2059		12q23-q24
ERBB2	2064	HER-2;HER2;NEU;NGL;TKR1	17q21.1

ERBB3	2065	HER3	12q13
ERBB4	2066	HER4	2q33.3-q34
ERCC1	2067	UV20	19q13.2-q13.3
ERCC2	2068	EM9;MAG;XPD	19q13.3
ERCC3	2071	BTF2;GTF2H;RAD25;TFIIH;XPB	2q21
ERCC4	2072	RAD1;XPF	16p13.3-p13.11
ERCC5	2073	ERCM2;UVDR;XPG;XPGC	13q33
ERCC6	2074	CKN2;COFS;CSB;RAD26	10q11
ERG	2078		21q22.3
ESR1	2099	ER;ESR;ESRA;Era;NR3A1	6q25.1
ETS1	2113		11q23.3
ETS2	2114		21q22.2
ETV1	2115	ER81	7p22
ETV6	2120	TEL	12p13
EVI1	2122	MDS1-EVI1;PRDM3	3q24-q28
EVI2A	2123	EVDA;EVI2	17q11.2
EXT1	2131	EXT	8q24.11-q24.13
EXT2	2132		11p12-p11
FANCA	2175	FA;FA-H;FA1;FAA;FACA;FAH;FANCH	16q24.3
FANCG	2189	FAG;XRCC9	9p13
FAT	2195	CDHF7;ME5	4q34-q35
FER	2241	TYK3	5q21
FES	2242	FPS	15q26.1
FGF1	2246	AFGF;ECGF;ECGF-beta;ECGFA;ECGFB;FGF-alpha;FGFA;GLIO703;HBGF1	5q31
FGF12	2257	FGF12B;FHF1	3q28
FGF2	2247	BFGF;FGFB;HBGH-2	4q26-q27
FGF3	2248	HBGF-3;INT2	11q13
FGF5	2250	FGF5S	4q21
FGF6	2251	HBGF-6;HST2	12p13
FGF7	2252	HBGF-7;KGF	15q15-q21.1
FGF8	2253	AIGF;HBGF-8	10q24
FGF9	2254	GAF;HBFG-9	13q11-q12
FGFR1	2260	BFGFR;C-FGR;CEK;FLG;FLJ14326;FLT2;H2;H3;H4;H5;KAL2;N-SAM	8p11.2-p11.1
FGFR2	2263	BEK;BFR-1;CEK3;CFD1;ECT1;JWS;K-SAM;KGFR;TK14;TK25	10q26
FGFR3	2261	ACH;CEK2;HSFGFR3EX;JTK4	4p16.3

FGFR4	2264	JTK2;TKF	5q35.1-qter
FGR	2268	SRC2;c-fgr;p55c-fgr	1p36.2-p36.1
FHIT	2272	AP3Aase;FRA3B	3p14.2
FLI1	2313	EWSR2;SIC-1	11q24.1-q24.3
FLT1	2321	FLT;VEGFR1	13q12
FLT3	2322	CD135;FLK2;STK1	13q12
FLT4	2324	PCL;VEGFR3	5q34-q35
FOLR1	2348	FBP;FOLR;FR-alpha;MOv18	11q13.3-q14.1
FOS	2353		14q24.3
FOSB	2354	GOS3;GOSB	19q13.32
FOSL2	2355	FRA2	2p23-p22
FRAP1	2475	FRAP;FRAP2;MTOR;RAFT1;RAPT1	1p36.2
FRZB	2487	FRP-3;FRZB-PEN;FRZB1;SFRP3	2qter
FVT1	2531		18q21.3
FYN	2534	MGC45350;SLK;SYN	6q21
FZD7	8324	FzE3	2q33
G22P1	2547	D22S671;D22S731;KU70;ML8;TLAA	22q13.2-q13.31
GADD45A	1647	DDIT1;GADD45	1p31.2-p31.1
GAS1	2619		9q21.3-q22
GAS7	8522	KIAA0394	17p
GFI1	2672	ZNF163	1p22
GLI	2735	GLI1	12q13.2-q13.3
GLI2	2736	THP2	2q14
GLI3	2737	GCPS;PAP-A;PAPA;PAPA1;PAPB;PHS;PPDIV	7p13
GML	2765	LY6DL	8q24.3
GRB2	2885		17q24-q25
GRB7	2886		17q21.2
GRPR	2925		Xp22.2-p22.13
GSTP1	2950	DFN7;FAEES3;GST3;PI	11q13
HCK	3055	JTK9	20q11-q12
HDAC1	3065	HD1;RPD3;RPD3L1	1p34
HDGF	3068	HMG1L2	Xq25
HIF1A	3091	HIF-1alpha;HIF1-ALPHA;MOP1	14q21-q24
HLF	3131		17q22
HMMR	3161	RHAMM	5q33.2-qter
HOXA9	3205	ABD-B;HOX1.7;HOX1G;MGC1934	7p15-p14
HRAS	3265	HRAS1;RASH1	11p15.5
ICAM1	3383	BB2;CD54	19p13.3-p13.2
IFNG	3458	IFG;IFI	12q14
IFNGR1	3459	CD119;IFNGR	6q23-q24
IFNGR2	3460	AF-1;IFGR2;IFNGT1	21q22.11
IGF1	3479	IGFI	12q22-q23
IGF1R	3480	JTK13	15q25-q26

IGF2	3481		11p15.5
IGF2R	3482	CD222;CIMPR;M6P-R;MPRI	6q26
IGFBP1	3484	IBP1;IGF-BP25	7p13-p12
IGFBP2	3485	IBP2;IGF-BP53	2q33-q34
IGFBP3	3486	IBP3	7p13-p12
IGFBP5	3488	IBP5	2q33-q36
IGFBP6	3489	IBP6	12q13
IL11	3589	AGIF;IL-11	19q13.3-q13.4
IL12A	3592	CLMF;IL-12A;NFSK;NKSF1	3p12-q13.2
IL12B	3593	CLMF;CLMF2;IL-12B;NKSF;NKSF2	5q31.1-q33.1
IL13	3596	ALRH;IL-13;P600	5q31
IL1A	3552	IL-1A;IL1;IL1-ALPHA;IL1F1	2q14
IL1B	3553	IL-1;IL1-BETA;IL1F2	2q14
IL1RN	3557	ICIL-1RA;IL1F3;IL1RA;IRAP;MGC10430	2q14.2
IL2	3558	IL-2;TCGF	4q26-q27
IL3	3562	IL-3;MCGF;MULTI-CSF	5q31.1
IL4	3565	BSF1;IL-4	5q31.1
IL6	3569	BSF2;HGF;HSF;IFNB2;IL-6	7p21
IL8	3576	3-10C;AMCF-I;CXCL8;GCP-1;GCP1;IL-8;K60;LECT;LUCT;LYNAP;MDNCF;MONAP;NAF;NAP-1;NAP1;SCYB8;TSG-1;b-ENAP	4q13-q21
ILK	3611	P59	11p15.5-p15.4
ING1	3621	p33ING1	13q34
INHA	3623		2q33-q36
IRF1	3659	IRF-1;MAR	5q31.1
ITGB1	3688	CD29;FNRB;GPIIA;MDF2;MSK12;VLAB	10p11.2
ITGB4	3691		17q11-qter
JAK2	3717		9p24
JUN	3725	AP1	1p32-p31
JUNB	3726		19p13.2
JUND	3727		19p13.2
KAI1	3732	4F9;C33;CD82;GR15;IA4;R2;SAR2;ST6	11p11.2
KDR	3791	FLK1;VEGFR;VEGFR2	4q11-q12
KIT	3815	CD117;PBT;SCFR	4q11-q12
KRAS2	3845	C-K-RAS;K-RAS2A;K-RAS2B;KI-RAS;RASK2	12p12.1
L1CAM	3897	CAML1;CD171;HSAS;HSAS1;MASA;MIC5;N-CAML1;S10;SPG1	Xq28

LAF4	3899	MLLT2-like	2q11.2-q12
LAMB1	3912		7q22
LCK	3932		1p34.3
LCN2	3934	NGAL	9q34
LIF	3976	CDF;D-FACTOR;HILDA	22q12.2
LIG1	3978		19q13.2-q13.3
LIG3	3980		17q11.2-q12
LIG4	3981		13q33-q34
LMO1	4004	RBTN1;RHOM1;TTG1	11p15
LMO2	4005	RBTN2;RBTNL1;RHOM2;<br>TTG2	11p13
LTA	4049	LT;TNFB;TNFSF1	6p21.3
LYN	4067	JTK8	8q13
MAD	4084	MXD1	2p13-p12
MADH2	4087	JV18;JV18-<br>1;MADR2;SMAD2	18q21.1
MADH4	4089	DPC4;JIP;SMAD4	18q21.1
MAF	4094		16q22-q23
MALT1	10892	DKFZp434L132;MLT;MLT<br>1	18q21
MAP3K8	1326	COT;EST;ESTF;TPL2;Tpl-<br>2;c-COT	10p12.1
MAPK10	5602	FLJ12099;JNK3;JNK3A;P<br>RKM10;p493F12;p54bSA<br>PK	4q22.1-q23
MAPK14	1432	CSBP1;CSBP2;CSPB1;E<br>XIP;Mxi2;PRKM14;PRKM<br>15;RK;SAPK2A;p38;p38A<br>LPHA	6p21.3-p21.2
MAS1	4142	MAS	6q25.3-q26
MATK	4145	CHK;CTK;DKFZp434N12<br>12;HHYLTK;HYL;HYLTK;<br>Lsk;MGC1708;MGC2101	19p13.3
MBD2	8932	DMTase;NY-CO-41	18q21
MCAM	4162	CD146;MUC18	11q23.3
MCC	4163		5q21-q22
MCF2	4168	DBL	Xq27
MCL1	4170	EAT;MCL1L;MCL1S;MGC<br>1839;TM	1q21
MDM4	4194	MDMX	1q32
MDS1	4197	MDS1-EVI1;PRDM3	3q26
MEL	4218	RAB8	19p13.1
MEN1	4221	MEAI;SCG2	11q13
MET	4233	HGFR;RCCP2	7q31
MLF1	4291		3q25.1
MLF2	8079		12p13
MLH1	4292	COCA2;FCC2;HNPCC;H<br>NPCC2;MGC5172;hMLH<br>1	3p21.3
MLL	4297	ALL-1;HRX;HTRX1;TRX1	11q23
MLLT3	4300	AF9	9p22

MLLT4	4301	AF-6;AF6;AFADIN	6q27
MLLT6	4302	AF17	17q21
MMP1	4312	CLG;CLGN	11q22.3
MMP10	4319	SL-2;STMY2	11q22.3
MMP14	4323	MMP-X1;MT1-MMP;MTMMP1	14q11-q12
MMP2	4313	CLG4;CLG4A;TBE-1	16q13-q21
MMP3	4314	SL-1;STMY;STMY1;STR1;TRANSIN	11q22.3
MMP7	4316	MMP-7;MPSL1;PUMP-1	11q21-q22
MMP9	4318	CLG4B;GELB	20q11.2-q13.1
MOS	4342	MSV	8q11
MPL	4352	C-MPL;CD110;MPLV;TPOR	1p34
MRE11A	4361	ATLD;HNGS1;MRE11;MRE11B	11q21
MSF	10801	AF17q25;KIAA0991;MSF1;PNUTL4;SEPT9;SINT1	17q25
MSH2	4436	COCA1;FCC1;HNPCC;HNPCC1	2p22-p21
MSH3	4437		5q11-q12
MSH6	2956	GTBP;HNPCC5	2p16
MST1R	4486	CDw136;RON	3p21.3
MTA1	9112		14q32.3
MTHFR	4524		1p36.3
MUC1	4582	CD227;EMA;PEM;PUM	1q21
MXI1	4601	MAD2;MXD2	10q24-q25
MYB	4602		6q22-q23
MYBL2	4605	BMYB;MGC15600	20q13.1
MYC	4609	c-Myc	8q24.12-q24.13
MYCL1	4610	LMYC;MYCL	1p34.2
MYCL2	4611		Xq22-q23
MYCN	4613	NMYC	2p24.1
NAT2	10	AAC2	8p22
NBS1	4683	AT-V1;AT-V2;ATV;NBS;NIBRIN	8q21
NEO1	4756	NGN	15q22.3-q23
NF1	4763	NFNS;VRNF;WSS	17q11.2
NFKB1	4790	EBP-1;KBF1;MGC54151;NF-kappa-B;NFKB-p105;NFKB-p50	4q24
NFKB2	4791	LYT-10;LYT10	10q24
NFKBIA	4792	IKBA;MAD-3;NFKBI	14q13
NGFR	4804	TNFRSF16;p75(NTR)	17q21-q22
NOS3	4846	ECNOS;eNOS	7q36
NOTCH1	4851	TAN1;hN1	9q34.3
NOTCH2	4853	hN2	1p13-p11
NOTCH4	4855	INT3;NOTCH3	6p21.3

NQO1	1728	DHQU;DIA4;DTD;NMOR1;NMORI;QR1	16q22.1
NRAS	4893	N-ras;NRAS1	1p13.2
NTRK1	4914	MTC;TRK;TRKA	1q21-q22
NTRK2	4915	TRKB	9q22.1
NTRK3	4916	TRKC	15q25
NUMA1	4926	NUMA	11q13
OGG1	4968	HMMH;HOGG1;MUTM;OGH1	3p26.2
OSM	5008	MGC20461	22q12.2
PBX1	5087		1q23
PCNA	5111	MGC8367	20pter-p12
PCTK1	5127	PCTAIRE1;PCTGAIRE	Xp11.3-p11.23
PDGFA	5154	PDGF1	7p22
PDGFB	5155	PDGF2;SIS;SSV;c-sis	22q13.1
PDGFRA	5156	CD140A;PDGFR2	4q11-q13
PDGFRB	5159	CD140B;JTK12;PDGF-R-beta;PDGFR;PDGFR1	5q31-q32
PGF	5228	PLGF;PlGF-2	14q24-q31
PGR	5241	NR3C3;PR	11q22-q23
PIK3CA	5290		3q26.3
PIM1	5292	PIM	6p21.2
PLA2G2A	5320	MOM1;PLA2B;PLA2L;PLA2S;PLAS1	1p35
PLAG1	5324		8q12
PLAT	5327	T-PA;TPA	8p12
PLAUR	5329	CD87;UPAR;URKR	19q13
PLG	5340		6q26
PML	5371	MYL;RNF71;TRIM19	15q22
PMS1	5378	HNPCC3;PMSL1;hPMS1	2q31.1
PNUTL1	5413	CDCREL;CDCREL-1;CDCREL1;H5;HCDCREL-1;SEPT5	22q11.21
PPARD	5467	FAAR;MGC3931;NR1C2;NUC1;NUCI;NUCII;PPAR-beta;PPARB	6p21.2-p21.1
PPARG	5468	HUMPPARG;NR1C3;PPARG1;PPARG2	3p25
PPP2R1B	5519	MGC26454	11q23.2
PRCC	5546	MGC17178;MGC4723;RCCP1;TPRC	1q21.1
PRKAR1A	5573	CAR;CNC1;PRKAR1;TSE1	17q23-q24
PRKR	5610	EIF2AK1;PKR	2p22-p21
PTCH	5727	BCNS;HPE7;NBCCS;PTC;PTC1	9q22.3
PTCH2	8643		1p33-p34
PTEN	5728	BZS;MGC11227;MHAM;MMAC1;PTEN1;TEP1	10q23.3

PTGS1	5742	COX1;COX3;PCOX1;PGG/HS;PGHS-1;PGHS1;PHS1;PTGHS	9q32-q33.3
PTGS2	5743	COX-2;COX2;PGG/HS;PGHS-2;PHS-2;hCox-2	1q25.2-q25.3
PTHLH	5744	HHM;MGC14611;PLP;PTHR;PTHRP	12p12.1-p11.2
PTK2	5747	FADK;FAK;FAK1;pp125FAK	8q24-qter
PTK7	5754	CCK4	6p21.1-p12.2
PTPRF	5792	LAR	1p34
PTPRG	5793	HPTPG;PTPG;R-PTP-GAMMA;RPTPG	3p21-p14
PTPRH	5794	SAP-1	19q13.4
PURA	5813	PUR-ALPHA;PUR1;PURALPHA	5q31
PXN	5829		12q24
QARS	5859	GLNRS	3p21.3-p21.1
RAD23A	5886	HHR23A	19p13.2
RAD50	10111	RAD50-2;hRad50	5q31
RAD51	5888	HRAD51;HsRad51;RAD51A;RECA	15q15.1
RAD52	5893		12p13-p12.2
RAD54B	25788		8q21.3-q22
RAD54L	8438	HR54;HRAD54;hHR54;hRAD54	1p32
RAF1	5894		3p25
RALB	5899		2cen-q13
RAN	5901	ARA24;TC4	6p21
RAP1A	5906	KREV-1;KREV1;SMGP21	1p13.3
RAP1GDS1	5910	GDS1	4q23-q25
RAP2A	5911	KREV;RAP2;RbBP-30	13q34
RARA	5914	NR1B1;RAR	17q21
RARB	5915	HAP;NR1B2;RRB2	3p24
RASA1	5921	CMAVM;GAP;PKWS;RASA;RASGAP;p120GAP	5q13.3
RB1	5925	RB	13q14.2
RBBP1	5926	RBP-1;RBP1	14q22.3-q23.1
RBBP2	5927	RBP2	12p11
RBBP5	5929	RBQ3	1q32
RBBP6	5930	DKFZp761B2423;MY038;RBQ-1	16p12.2
RBL2	5934	P130;Rb2	16q12.2
RECQL	5965	RECQL1;RecQ1	12p12
REL	5966	C-Rel	2p13-p12
RELA	5970	NFKB3	11q13

RET	5979	CDHF12;HSCR1;MEN2A;MEN2B;MTC1;PTC;RET51	10q11.2
RIPK1	8737	RIP	6p25.2
RLF	6018		1p32
ROS1	6098	MCF3;ROS	6q22
RRAS	6237		19q13.3-qter
S100A4	6275	18A2;42A;CAPL;MTS1;P9KA;PEL98	1q21
SEMA3F	6405	SEMA-IV;SEMA4;SEMAK;semaIV	3p21.3
SERPINE1	5054	PAI;PAI-1;PAI1;PLANH1	7q21.3-q22
SH3BP2	6452	CRBM;CRPM;RES4-23	4p16.3
SHH	6469	HHG1;HLP3;HPE3;SMMCI	7q36
SIAH1	6477		16q12
SKI	6497	SKV	1q22-q24
SKIL	6498	SNO	3q26
SMARCA4	6597	BAF190;BRG1;SNF2-BETA;SNF2L4;SNF2LB;hSNF2B;hSNF2b	19p13.2
SMARCB1	6598	BAF47;INI1;RDT;SNF5;SNF5L1;Sfh1p;Snr1;hSNFS	22q11
SOD1	6647	ALS;ALS1;IPOA	21q22.11
SPARC	6678	ON	5q31.3-q32
SPI1	6688	OF;PU.1	11p11.2
SPP1	6696	BNSP;BSPI;ETA-1;OPN	4q21-q25
SRC	6714	ASV;SRC1;c-SRC;p60-Src	20q12-q13
STAT1	6772	ISGF-3;STAT91	2q32.2
STAT3	6774	APRF	17q21
STAT5B	6777	STAT5	17q11.2
STK11	6794	LKB1;PJS	19p13.3
SYK	6850		9q22
TAL1	6886	SCL;TCL5	1p32
TCF7L2	6934	TCF-4;TCF4	10q25.3
TDGF1	6997	CR;CRGF;CRIPTO	3p21.31
TEK	7010	CD202B;TIE-2;TIE2;VMCM;VMCM1	9p21
TERT	7015	EST2;TCS1;TP2;TRT;hEST2	5p15.33
TFAP2C	7022	AP2-GAMMA;ERF1	20q13.2
TFDP1	7027	DP1;DRTF1;Dp-1	13q34
TFE3	7030		Xp11.22
TFF1	7031	BCEI;D21S21;HPS2;pNR-2;pS2	21q22.3
TFG	10342	TF6	3q11-q12
TFRC	7037	CD71;TFR;TRFR	3q26.2-qter
TGFA	7039		2p13

TGFB1	7040	CED;DPD1;TGFB	19q13.1
TGFB2	7042		1q41
TGFB3	7043		14q24
TGFBI	7045	BIGH3;CDB1;CDGG1;CSD;CSD1;CSD2;CSD3;LCD1	5q31
TGFBR1	7046	ACVRLK4;ALK-5	9q22
TGFBR2	7048	HNPCC6	3p22
TGFBR3	7049		1p33-p32
THBS2	7058	TSP2	6q27
THPO	7066	MGDF;MKCSF;ML;MPLLG;TPO	3q27
TIAM1	7074		21q22.11
TIMP1	7076	CLGI;EPA;EPO;HCI;TIMP	Xp11.3-p11.23
TIMP2	7077		17q25
TIMP3	7078	HSMRK222;K222TA2;SFD	22q12.3
TK1	7083		17q23.2-q25.3
TNF	7124	CACHECTIN;DIF;TNFA;TNFSF2	6p21.3
TNFAIP1	7126	B12;B61;EDP1;MGC2317	17q22-q23
TNFRSF10A	8797	APO2;DR4;MGC9365;TRAILR-1;TRAILR1	8p21
TNFRSF10B	8795	DR5;KILLER;KILLER/DR5;TRAIL-R2;TRAILR2;TRICK2;TRICK2A;TRICK2B;TRICKB;ZTNFR9	8p22-p21
TNFRSF1A	7132	CD120a;FPF;MGC19588;TBP1;TNF-R;TNF-R-I;TNF-R55;TNFAR;TNFR1;TNFR55;TNFR60;p55;p55-R;p60	12p13.2
TNFRSF1B	7133	CD120b;TBPII;TNF-R-II;TNF-R75;TNFBR;TNFR2;TNFR80;p75;p75TNFR	1p36.3-p36.2
TNFRSF5	958	Bp50;CD40;CDW40;MGC9013;p50	20q12-q13.2
TNFRSF6	355	APO-1;APT1;CD95;FAS;FASTM	10q24.1
TNFSF10	8743	APO2L;Apo-2L;TL2;TRAIL	3q26
TNFSF6	356	APT1LG1;CD178;CD95L;FASL	1q23
TNFSF8	944	CD153;CD30L;CD30LG	9q33
TOP1	7150	TOPI	20q12-q13.1
TOP2A	7153	TOP2;TP2A	17q21-q22

TP53	7157	P53;TRP53;p53	17p13.1
TP73	7161	P73	1p36.3
TPR	7175		1q25
TRAF3	7187	CAP-1;CD40bp;CRAF1;LAP1	14q32.33
TRAF4	9618	CART1;MLN62;RNF83	17q11-q12
TSC1	7248	HAMARTIN;KIAA0243;LAM;TSC	9q34
TSC2	7249	LAM;TUBERIN	16p13.3
TSG101	7251	TSG10	11p15
TYMS	7298	HsT422;TMS;TS;TSase	18p11.32
TYRO3	7301	BYK;RSE	15q15.1-q21.1
VAV1	7409	VAV	19p13.2
VAV2	7410		9q34.1
VBP1	7411	PFD3;PFDN3;VBP-1	Xq28
VEGF	7422	VEGFA	6p12
VEGFB	7423	VEGFL;VRF	11q13
VHL	7428		3p26-p25
VIL2	7430	CVIL;CVL;DKFZp762H157;MGC1584	6q25.2-q26
WEE1	7465	WEE1hu	11p15.3-p15.1
WNT1	7471	INT1	12q13
WNT10B	7480	WNT-12	12q13
WNT2	7472	INT1L1;IRP	7q31
WNT2B	7482	WNT13;XWNT2	1p13
WNT5A	7474	hWNT5A	3p21-p14
WNT8B	7479		10q24
WRN	7486	RECQ3;RECQL2;RECQL3	8p12-p11.2
WT1	7490	GUD;WAGR;WIT-2;WT33	11p13
XPA	7507	XP1;XPAC	9q22.3
XPC	7508	XP3;XPCC	3p25
XRCC1	7515	RCC	19q13.2
XRCC2	7516		7q36.1
XRCC4	7518		5q13-q14
XRCC5	7520	KARP-1;KARP1;KU80;Ku86;NFIV	2q35
YES1	7525	C-YES;HsT441;P61-YES;Yes;c-yes	18p11.31-p11.21
YY1	7528	DELTA;NF-E1;UCRBP;YIN-YANG-1	14q
ZNF146	7705	OZF	19q13.1
ZNFN1A1	10320	Hs.54452;IK1;IKAROS;LYF1;PRO0758;hIk-1	7p13-p11.1
