# Supplementary material for: Wnt signaling in triple negative breast cancer is associated with metastasis
Source: BMC Cancer. 2013 Nov 10;13:537. doi: 10.1186/1471-2407-13-537 (PMC4226307; doi:10.1186/1471-2407-13-537)
Supplement: Additional file 2: Table S2 — Custom Cancer Panel. [file 1471-2407-13-537-S2.pdf]

S1: DASL Assay Reproducibility and Concordance

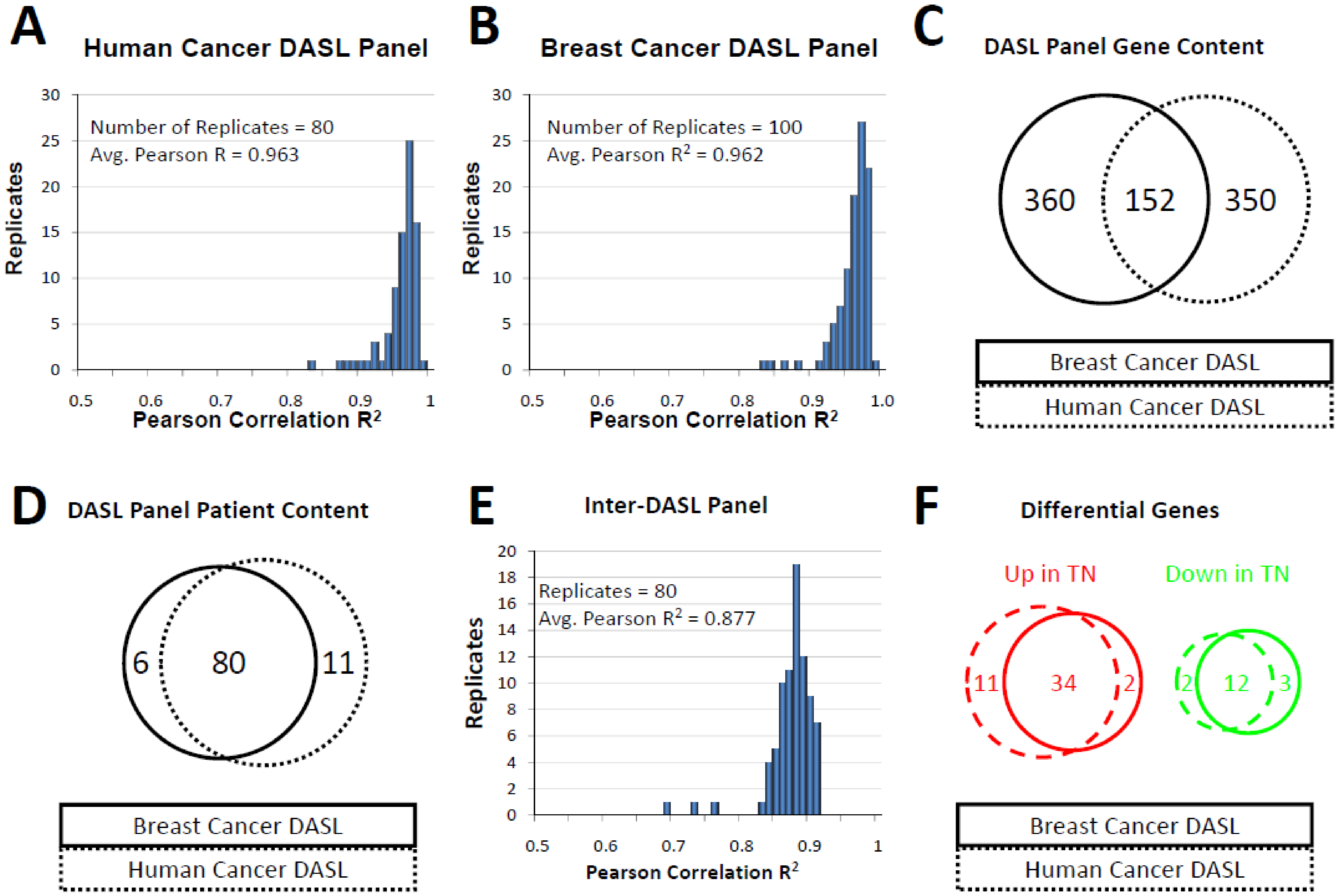

S1: DASL Assay Reproducibility and Concordance

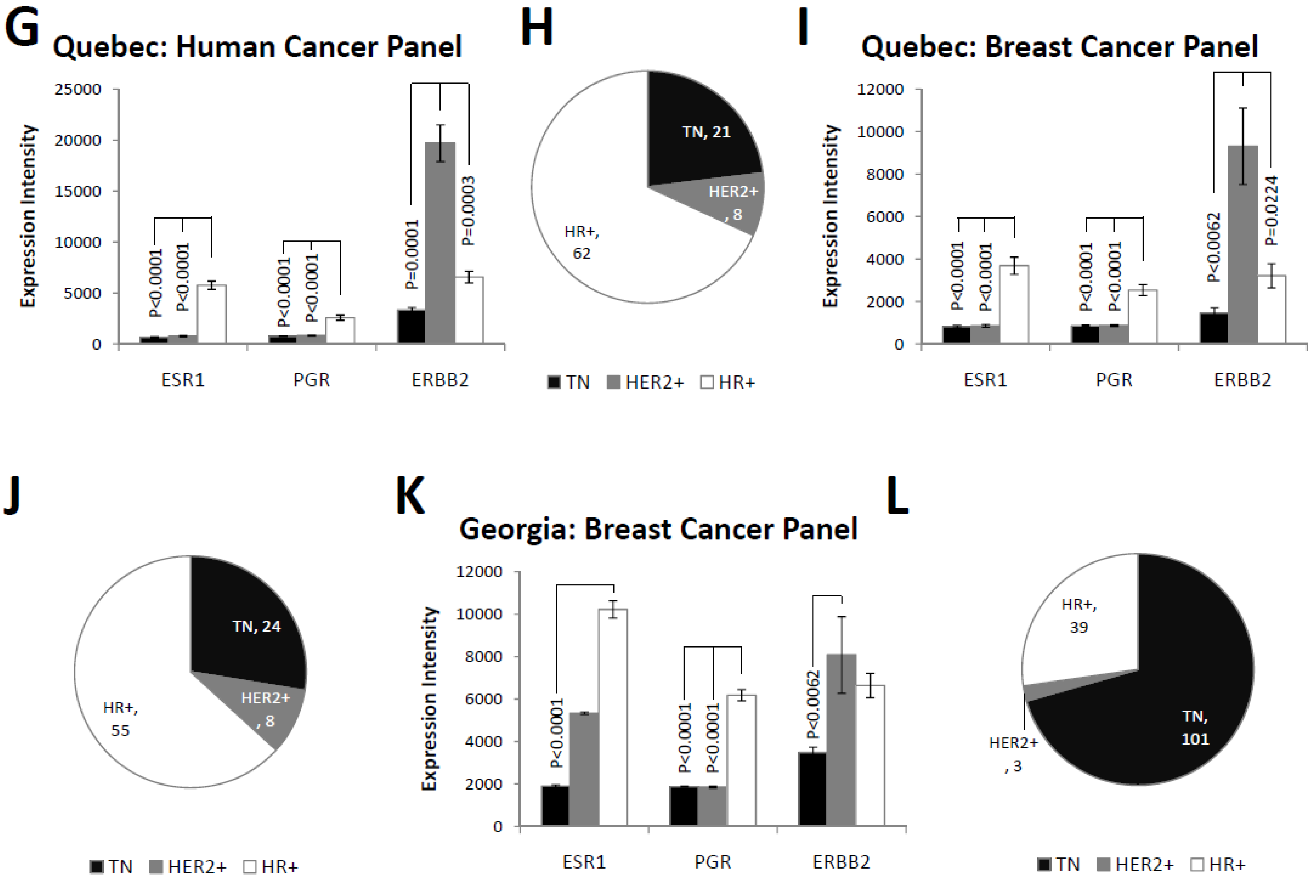

S2: Canonical Pathways Overrepresented in TN / Basal-like Subtypes

A — QC-HCP

Pathway	Probes total	Probes up in TN	P	corr. P
ErbB	99	7	0.5125	1
Hedgehog	42	1	0.9565	1
JAK-STAT	103	3	0.977	1
MAPK	195	10	0.811	1
mTOR	39	0	1	1
Notch	18	1	0.7426	1
PI3K	6	0	1	1
TGF-β	58	1	0.9864	1
VEGF	51	0	1	1
Wnt	77	5	0.6246	1
Total	1488	89	-	-

B — QC-BCP

Pathway	Probes total	Probes up in TN	P	corr. P
ErbB	75	6	0.3709	1
Hedgehog	6	0	1	1
JAK-STAT	63	2	0.9292	1
MAPK	111	8	0.4485	1
mTOR	45	0	1	1
Notch	18	0	1	1
PI3K	9	0	1	1
TGF-β	39	2	0.74	1
VEGF	48	0	1	1
Wnt	42	8	0.0048	0.0481
Total	1536	101	-	-

C — GA-BCP

Pathway	Probes total	Probes up in TN	P	corr. P
ErbB	75	5	0.9612	1
Hedgehog	6	0	1	1
JAK-STAT	63	9	0.3506	1
MAPK	111	7	0.9877	1
mTOR	45	2	0.8571	1
Notch	18	0	1	1
PI3K	9	0	1	1
TGF-β	39	2	0.9603	1
VEGF	48	1	0.9982	1
Wnt	42	7	0.239	1
Total	1536	186	-	-

D — MSKCC-99

Pathway	Probes total	Probes up in TN	P	corr. P
ErbB	164	14	0.0768	0.7683
Hedgehog	77	9	0.0278	0.2783
JAK-STAT	251	20	0.0719	0.7185
MAPK	486	23	0.8261	1
mTOR	89	9	0.0611	0.6112
Notch	79	2	0.9404	1
PI3K	123	5	0.8253	1
TGF-β	147	10	0.3094	1
VEGF	129	13	0.0289	0.2889
Wnt	257	18	0.195	1
Total	22283	1248	-	-

E — UNCCH-186

Pathway	Probes total	Probes up in Basal	P	corr. P
ErbB	152	12	0.4943	1
Hedgehog	91	13	0.0204	0.2042
JAK-STAT	223	13	0.8765	1
MAPK	400	26	0.8292	1
mTOR	83	4	0.8862	1
Notch	57	2	0.9382	1
PI3K	119	7	0.8116	1
TGF-β	135	15	0.0907	0.9068
VEGF	122	8	0.7214	1
Wnt	270	32	0.0087	0.0869
Total	22575	1722	-	-

F — Stockholm-159

Pathway	Probes total	Probes up in Basal	P	corr. P
ErbB	164	6	0.1257	1
Hedgehog	77	4	0.0755	0.755
JAK-STAT	251	2	0.9675	1
MAPK	486	11	0.4239	1
mTOR	89	0	1	1
Notch	79	0	1	1
PI3K	123	5	0.1122	1
TGF-β	147	9	0.0036	0.0359
VEGF	129	3	0.5009	1
Wnt	257	16	0.0001	0.0010
Total	22283	461	-	-

S3: Canonical Pathway Regulation by Subtype

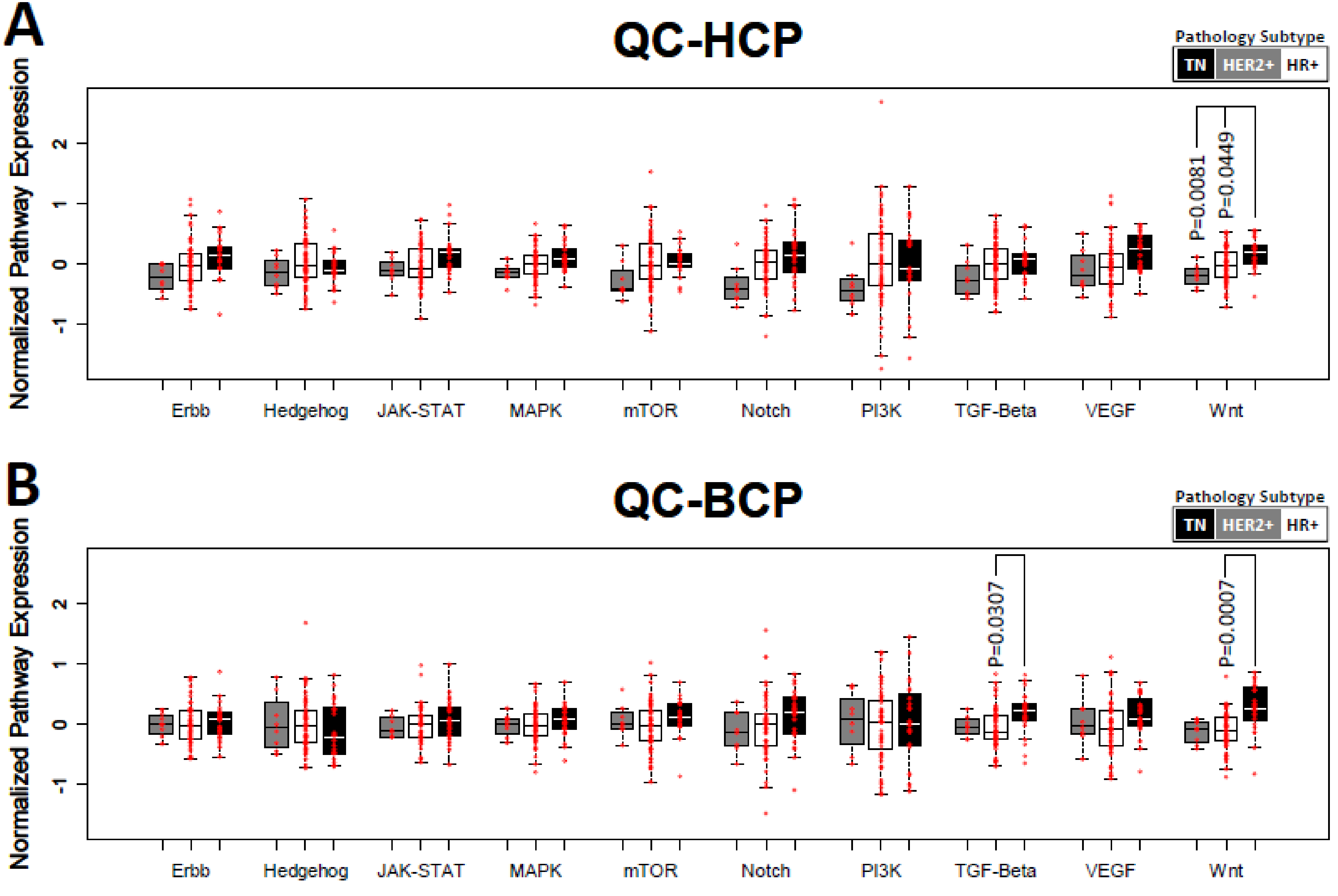

S3: Canonical Pathway Regulation by Subtype

C

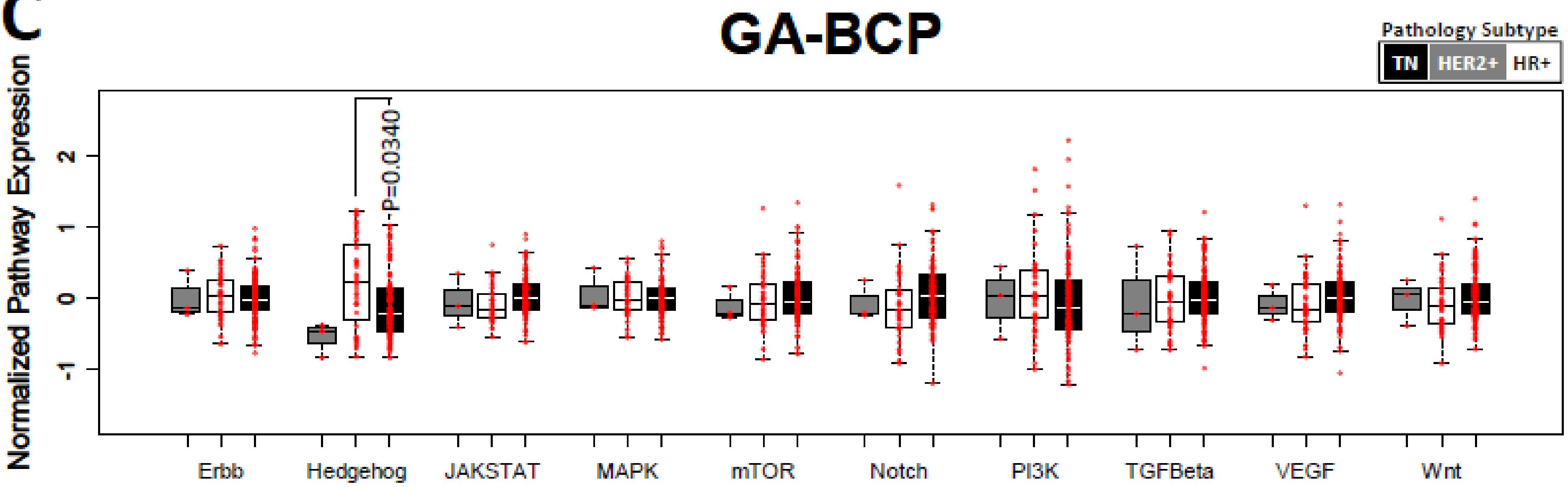

D

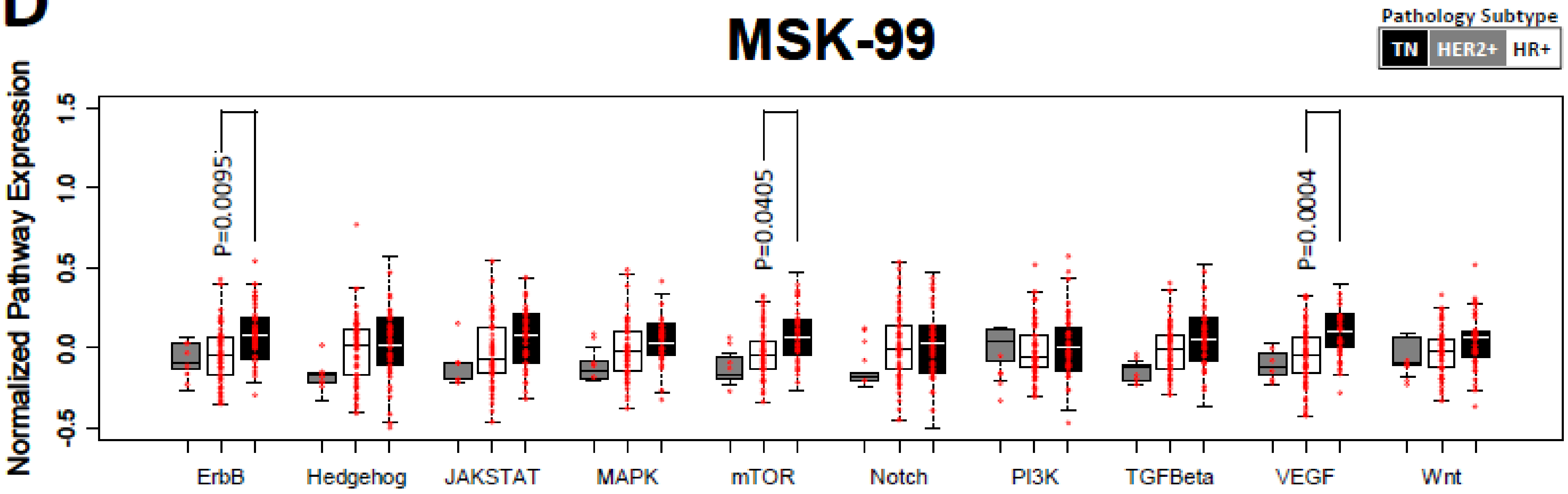

S3: Canonical Pathway Regulation by Subtype

E

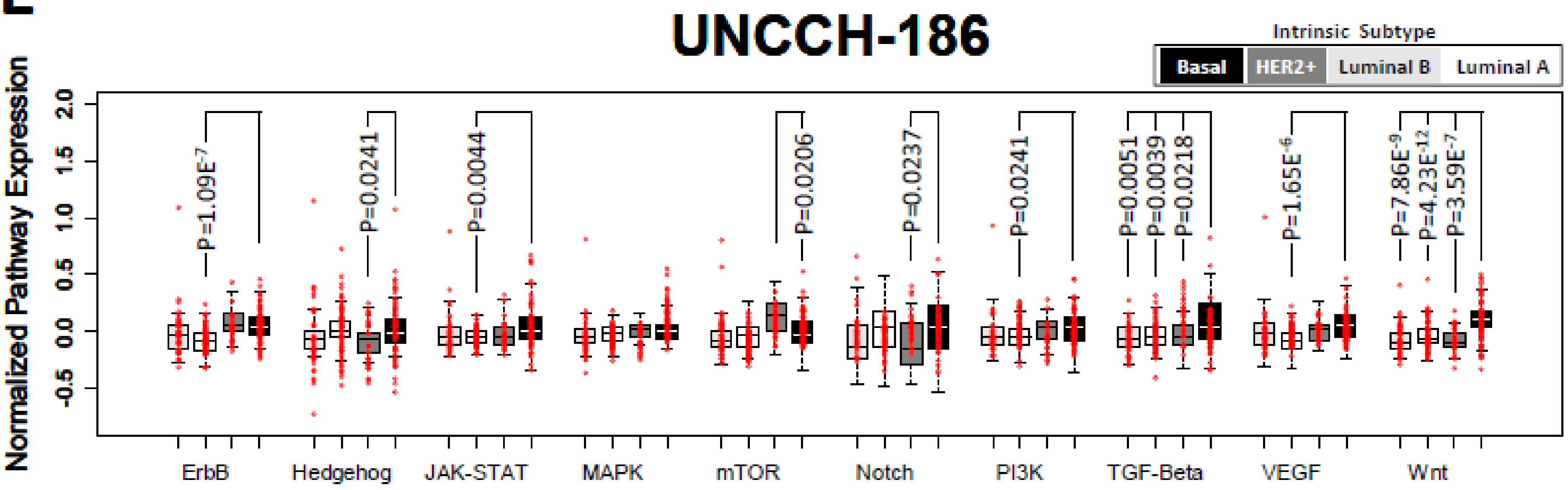

F

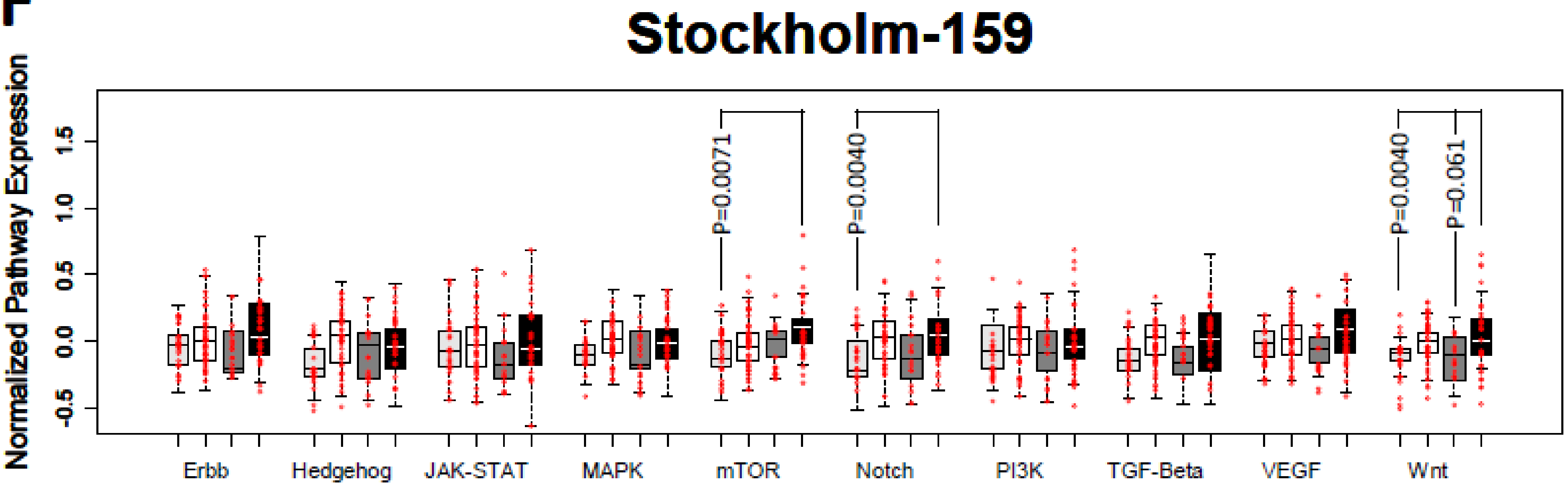

S4: KEGG Wnt Pathway Expression by Subtype

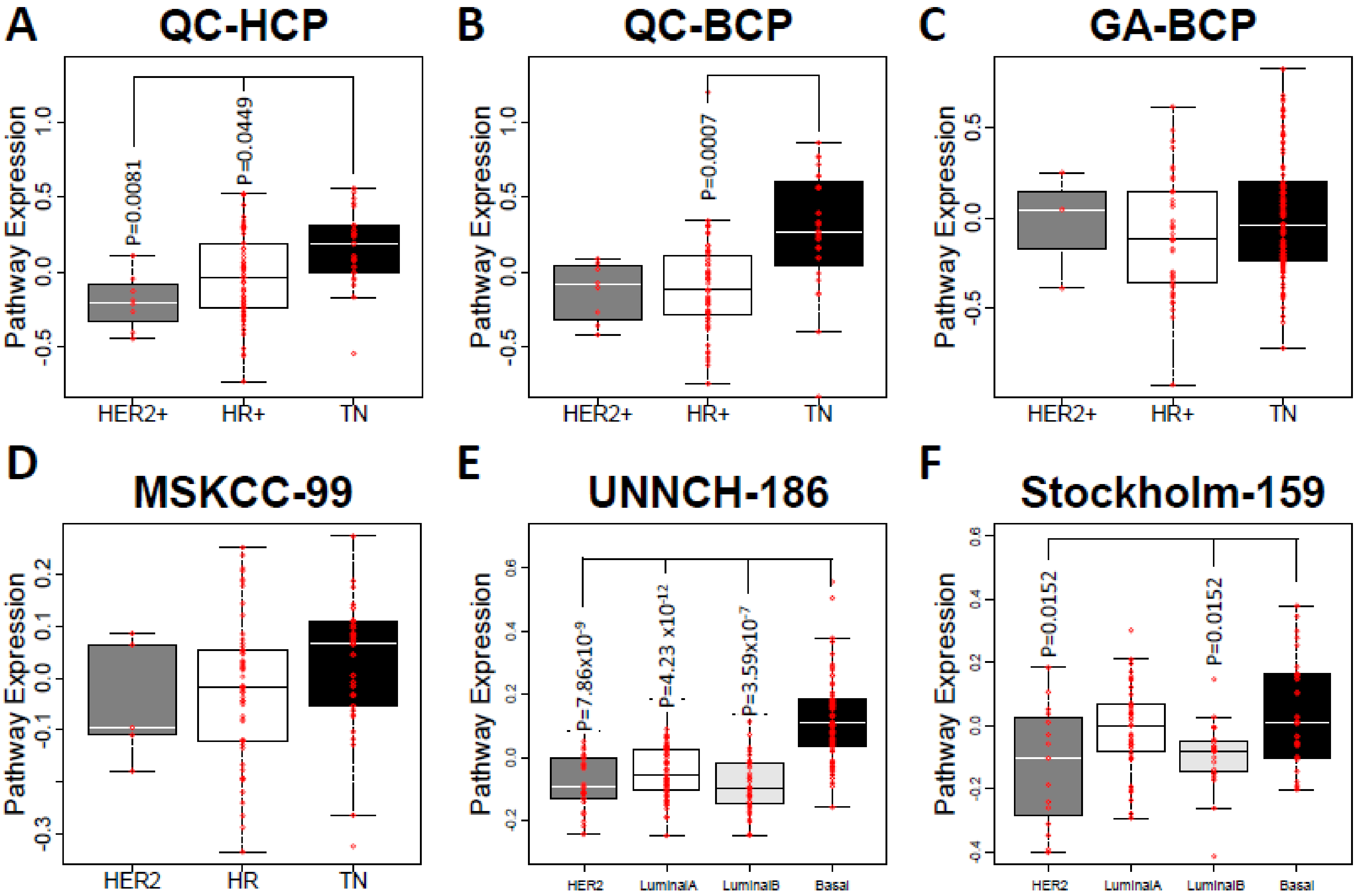

S5: Experimental Pathway Regulation by Subtype

A — QC-HCP

	Pathway Expression			Corrected P (v TN)	
Pathway	TN	HER2+	HR+	HER2+	HR+
β-catenin	0.49	0.09	-0.18	0.664	**7.7E-05**
E2F3	0.13	-0.26	-0.01	**0.011**	0.081
Myc	0.18	-0.15	-0.04	0.061	**0.005**
Ras	0.00	0.06	-0.01	0.292	1
Src	0.10	-0.23	0.00	0.124	0.741

B — QC-BCP

	Pathway Expression			Corrected P (v TN)	
Pathway	TN	HER2+	HR+	HER2+	HR+
β-catenin	0.59	0.02	-0.26	0.482	**8.8E-06**
E2F3	0.04	-0.08	-0.01	1	1
Myc	0.20	-0.03	-0.08	0.470	**0.014**
Ras	-0.01	0.03	0.00	0.845	0.987
Src	0.23	0.14	-0.12	1	0.119

C — GA-BCP

	Pathway Expression			Corrected P (v TN)	
Pathway	TN	HER2+	HR+	HER2+	HR+
β-catenin	0.16	-0.13	-0.40	1	**2.9E-04**
E2F3	0.05	-0.10	-0.12	0.871	**0.002**
Myc	0.09	0.07	-0.24	1	**5E-10**
Ras	0.00	0.00	0.01	1	0.383
Src	-0.01	0.19	0.00	1	1

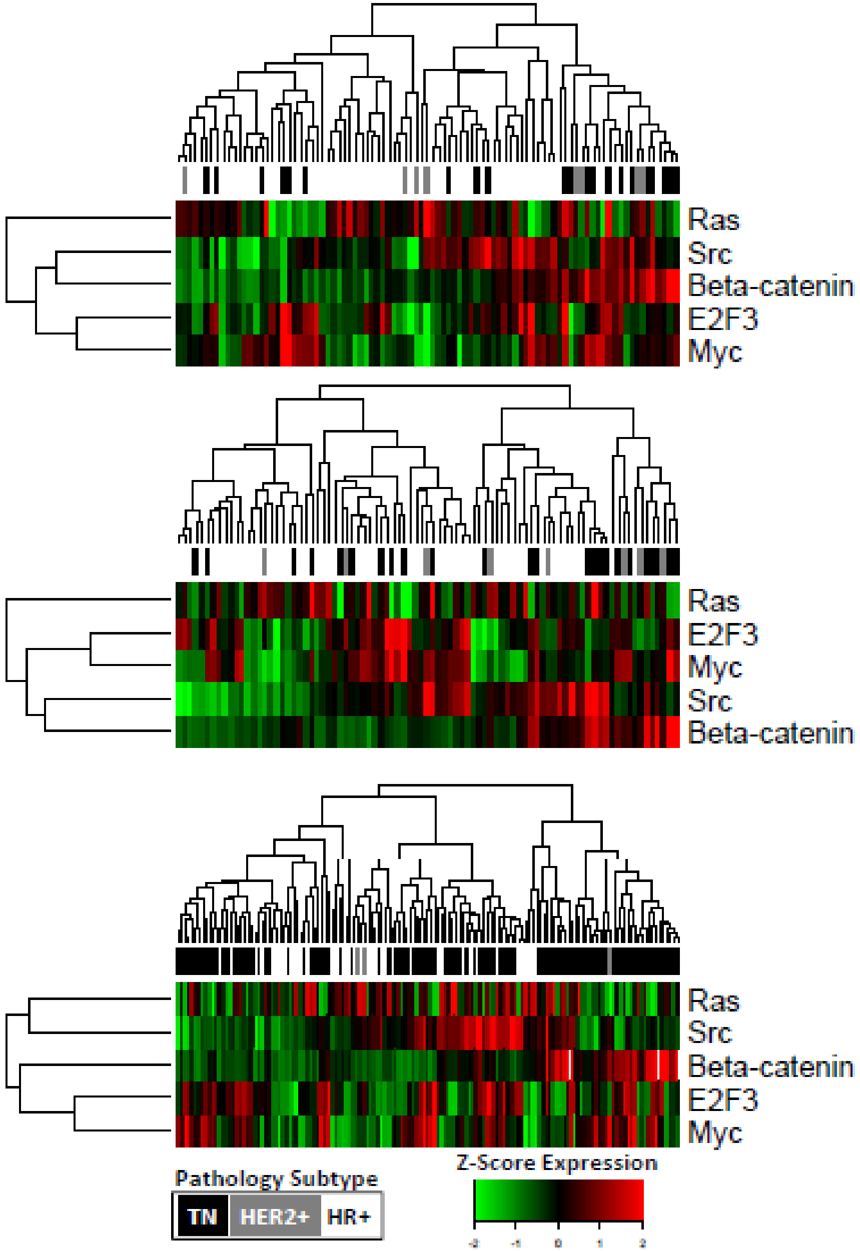

S5: Experimental Pathway Regulation by Subtype

D

MSKCC-99

Pathway	Pathway Expression			Corrected P (v TN)	
	TN	HER2+	HR+	HER2+	HR+
β-catenin	0.27	-0.16	-0.04	0.310	2E-08
E2F3	0.02	-0.01	-0.06	0.314	0.495
Myc	0.11	-0.07	-0.01	0.743	2.7E-05
Ras	0.02	-0.01	0.02	1	0.014
Src	0.09	-0.04	-0.17	0.028	0.024

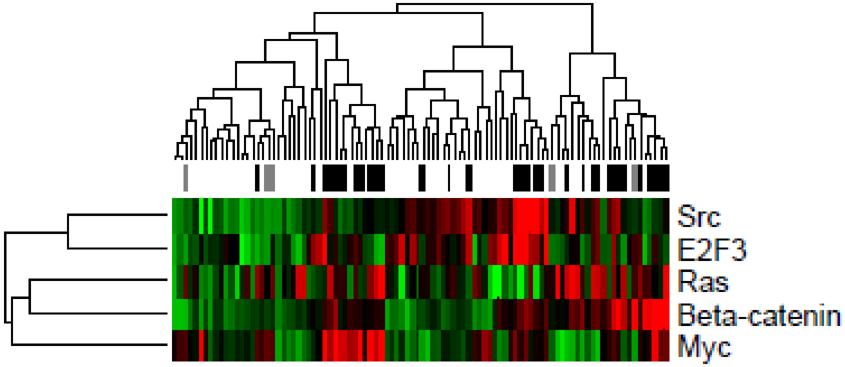

E

UNCCH-186

Pathway	Pathway Expression				Corrected P (v Basal)		
	Basal	HER2+	LumA	LumB	HER2+	LumA	LumB
β-catenin	0.20	-0.04	-0.07	-0.17	0.002	1E-05	2E-05
E2F3	0.05	-0.02	-0.05	0.03	0.005	7.8E-06	0.970
Myc	0.21	-0.07	-0.16	0.04	6.6E-09	1.1E-18	0.001
Ras	0.01	0.03	-0.01	-0.03	2.1E-02	0.195	0.001
Src	0.05	-0.08	0.02	-0.07	0.004	0.871	7.5E-03

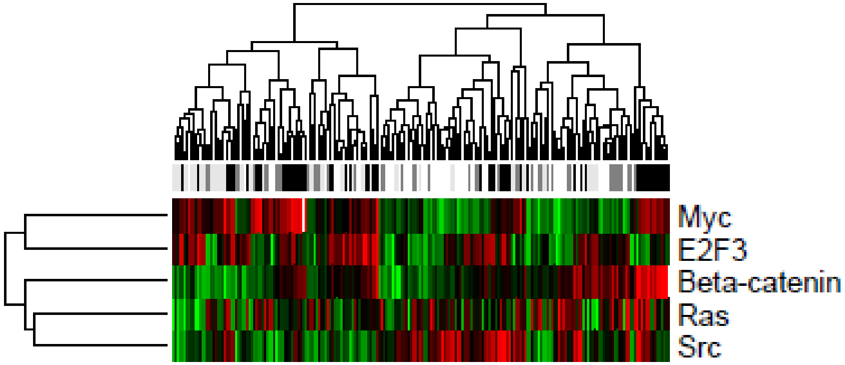

F

Stockholm-159

Pathway	Pathway Expression				Corrected P (v Basal)		
	Basal	HER2+	LumA	LumB	HER2+	LumA	LumB
β-catenin	0.33	-0.08	-0.10	-0.19	7.5E-03	1.3E-04	4.1E-05
E2F3	0.09	-0.08	0.00	-0.05	0.002	0.028	0.003
Myc	0.23	-0.03	-0.06	0.09	0.014	7.3E-06	0.169
Ras	0.01	0.05	-0.01	0.025	0.365	0.476	1
Src	0.01	-0.06	-0	-0.03	0.827	1	1

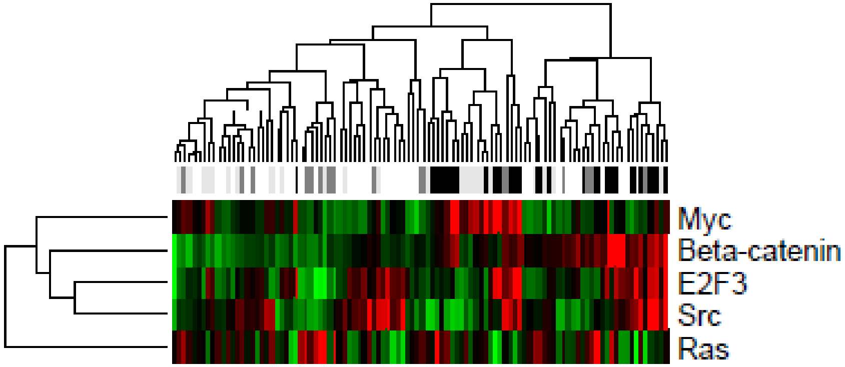

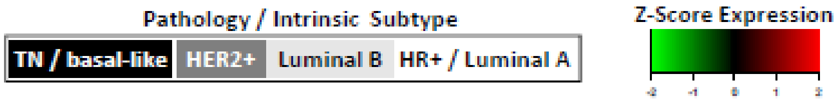

S6: Experimental Wnt/β-catenin Expression by Subtype

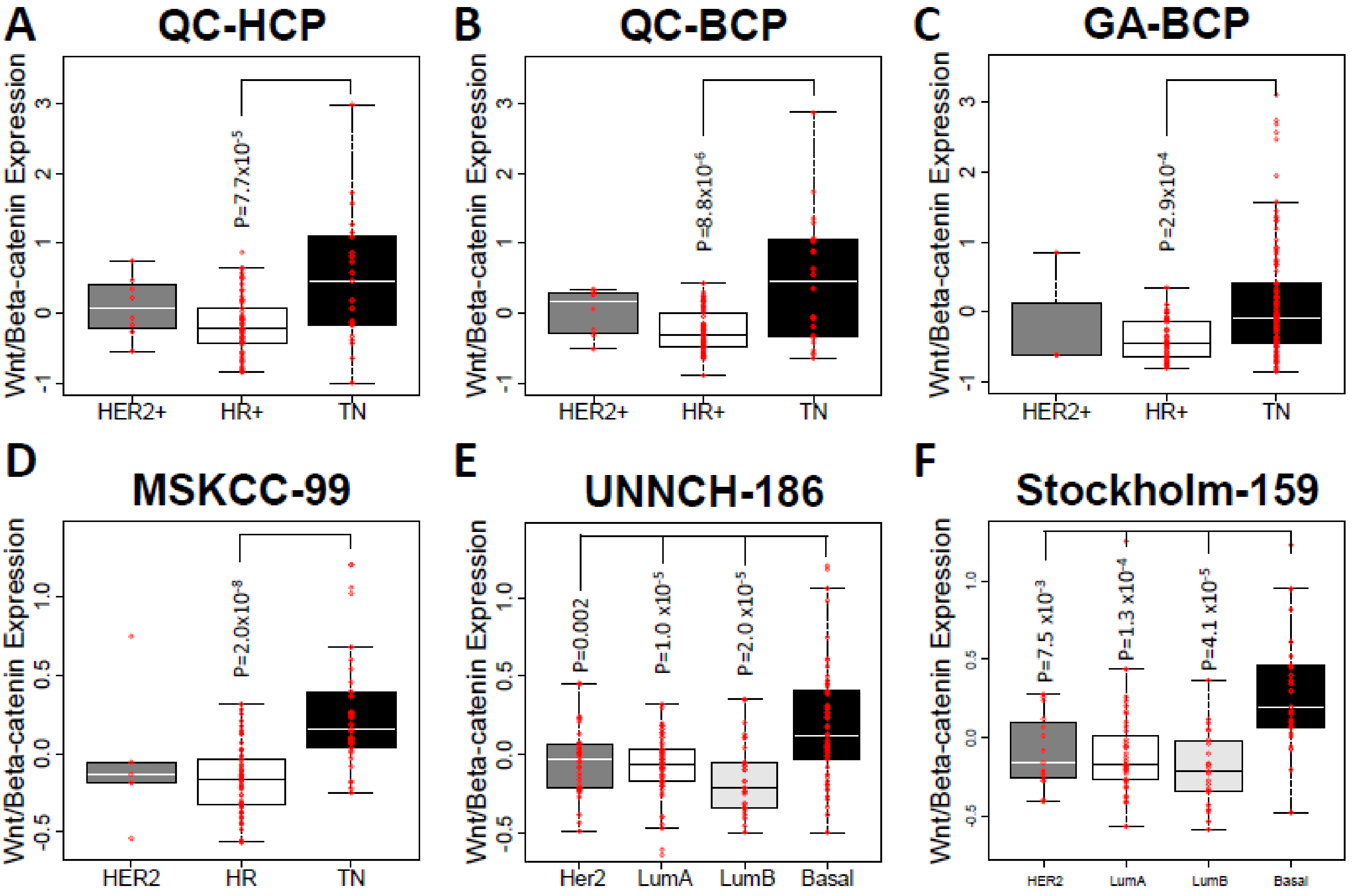

S7: Wnt/β-catenin Expression by Classifier Type

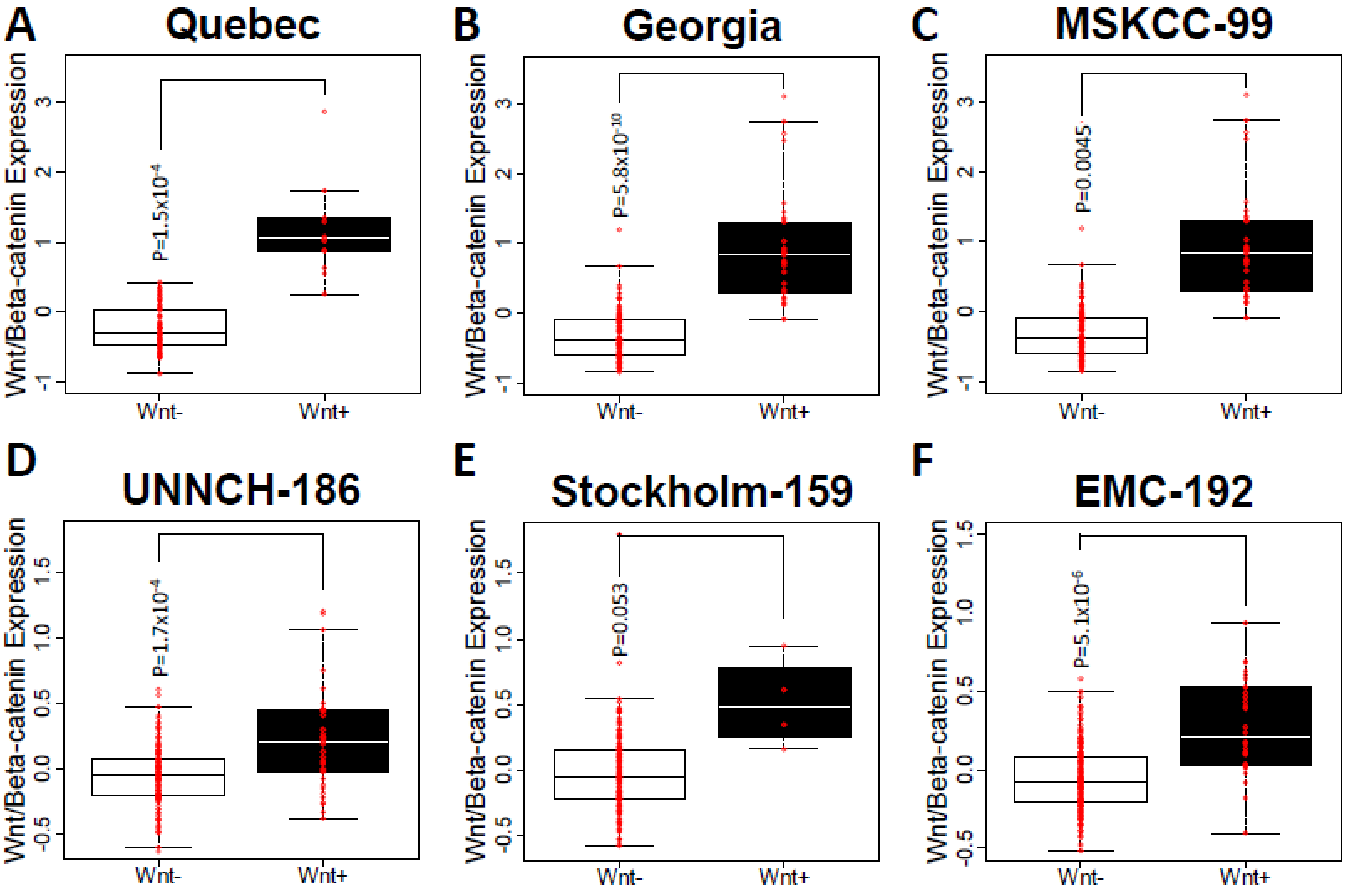

S7: Wnt/β-catenin Expression by Classifier Type

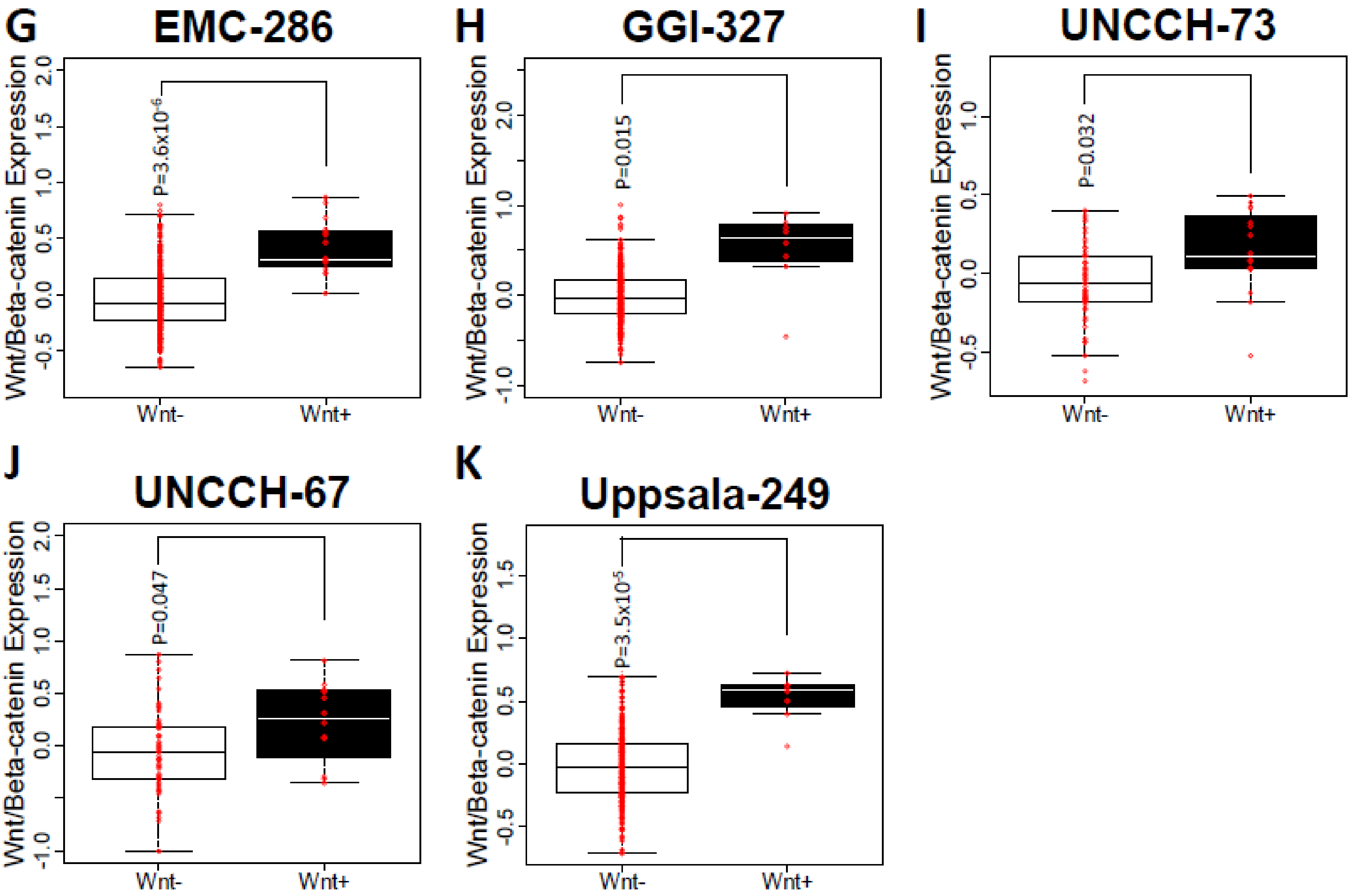

Table S3: Wnt+ Patients in Meta-Analysis

Meta-Analysis															
			Intrinsic Subtype					Path Subtype			Grade			Node	
Data Set	GEO	Size	Basal	HER2	LumA	LumB	Normal	HER2+	HR+	TN	1	2	3	P	N
EMC-192	GSE12276	192	-	-	-	-	-	-	-	-	-	-	-	-	-
EMC-286	GSE2034	286	-	-	-	-	-	-	-	-	-	-	-	0	286
Georgia	GSE18539	143	-	-	-	-	-	3	39	101	-	-	-	-	-
GGI-327	GSE6532	327	-	-	-	-	-	-	-	-	65	145	60	85	221
MSKCC-99	GSE2603	99	-	-	-	-	-	4	47	30	-	-	-	54	27
Quebec	GSE17650	97	-	-	-	-	-	8	54	24	-	-	-	-	-
STH-159	GSE1456	159	25	15	39	23	37	-	-	-	28	51	31	-	-
UNCCH-186	GSE10886	186	54	36	62	31	3	-	-	-	9	45	120	82	91
UNCCH-67	GSE6128	67	22	7	13	16	9	-	-	-	4	11	32	29	21
UNCCH-73	GSE3165	73	18	9	22	20	4	-	-	-	3	27	32	39	27
Uppsala-249	GSE4922	249	-	-	-	-	-	-	-	-	68	126	55	81	159
Wnt+		188	53	8	7	0	3	2	2	52	2	17	52	38	59
Wnt-		1690	66	59	129	90	50	13	138	103	175	388	278	332	773
Total		1878	119	67	136	90	53	15	140	155	177	405	330	370	832
Overrepresentation (p)			2.20E-16	0.84	1	1	0.99	0.79	1	6.30E-14	1	1	3.98E-11	0.042	0.97
