# Supplementary material for: Wnt signaling in triple negative breast cancer is associated with metastasis
Source: BMC Cancer. 2013 Nov 10;13:537. doi: 10.1186/1471-2407-13-537 (PMC4226307; doi:10.1186/1471-2407-13-537)
Supplement: Additional file 3 — Figure S1-Table S3: Assay reproducibility in the Quebec cohort measured by a distribution of Pearson R2 coefficients between RNA replicates. Figure S2: Analysis of upregulated probes in TNBC as compared to other subtypes in context with KEGG [21] signal transduction pathways. P-values were Bonferroni corrected and significant pathways (p <0.05) are in bold. Figure S3: Pathway expression of canonical KEGG signal transduction pathways [21] were measured as the normalized mean of the pathway components and subsequently used to calculate pathway perturbation between BC subtypes (see Additional file 4). Figure S4: Canonical Wnt expression of KEGG [21] pathway components. Significant p-values after Bonferroni’s correction are shown relative to the TNBC subtypes (see Additional file 4). Figure S5: Pathway expression of experimentally derived oncogenic signaling pathways from Bild et al. [22] measured between BC subtypes (see Additional file 4). Hierarchical clustering of pathway expression depicts patterns in pathway regulation (rows) in context with BC subtype (columns). Figure S6: Experimentally induced Wnt/β-catenin pathway expression from Bild et al. [22]. Significant p-values after Bonferroni’s correction are shown relative to the TNBC subtypes. Figure S7: Expression of Wnt/β-catenin components in patients classified as Wnt compared to Wnt + (Wnt classifier signature) in each of the 11 studies analyzed in the meta-analysis shows greater expression of tumors classified as Wnt + (Wnt classifier signature). Table S3: Table identifying the number of patients from each cohort in a meta-analysis of 11 studies and 1,878 patients with pathological or intrinsic determined subtype, grade, and lymph node status. Each category is broken down by total number of Wnt + (Wnt classifier signature) and Wnt- patients identified by the Wnt/β-catenin classifier and then analyzed for overrepresentation using Fisher’s exact test. [file 1471-2407-13-537-S3.pdf]

Supplementary Analysis and Statistical Methods

Analysis of Quebec and Georgia Microarray Data

Illumina DASL transcript intensities were interpreted in GenomeStudio, and data were quantile normalized and scaled per processing batch. Average, rank invariant, and cubic spline normalization methods were considered, but yielded lower Pearson R^2 coefficients between RNA replicates and ruled out on this basis. Multiple RNA replicates were mean combined to assess transcript expression per patient/tumor and data were further analyzed on this basis.

Differential Analysis

Differentially regulated probes were computed using permutation testing in the R package "samr" (1) where 500 permutations determined a false discovery rate (FDR) less than 1% in conjunction with a minimum fold-change of 1.5-fold difference in expression.

Hierarchical Clustering

Hierarchically clustered heatmaps were generated in R/Bioconductor (2) using the "heatmap.2" function of the "gplots" package with a Euclidean distance dissimilarity metric and an average linkage clustering algorithm. Heatmaps used probe-level data which were normalized (Z-score) for visualization purposes.

KEGG Pathways

KEGG signal transduction pathways were obtained from the KEGG database (http://www.genome.jp/kegg/) (3) and mapped via Entrez gene ID. Overrepresented KEGG pathways were assessed by Fisher's exact test. Two by two tables were built using the number of transcripts upregulated in the TN subtype and in a given pathway compared to non-upregulated transcripts and non-pathway transcripts (see example below). Fisher's exact test was calculated using the R/Bioconductor (2)"fisher.test" function of the "stats" package. P-values were corrected for multiple hypothesis testing using Bonferroni's correction for KEGG signal transduction pathways. Pathway expressions were calculated as the mean of the normalized (Z-score) gene components for each KEGG pathway, where each cohort analyzed with separate normalization for each cohort analyzed. Differentially expressed pathways between breast cancer subtypes were calculated using permutation testing in the R/Bioconductor package "samr" (1) with a FDR less than 1%, in conjunction with a t-statistic determined p-value corrected for multiple hypothesis test using Bonferroni's correction.

**QC-BCP**	**Wnt**	**Non-Wnt**	**Total**	
**Upregulated in TN**	8	93	101	**P = 0.00481**
**Not upregulated in TN**	34	1401	1435	
**Total**	42	1494	1536	

HMEC Oncogenic Pathways

Data for adenoviral transfected Human Mammary Epithelial Cells (HMECs) published by Bild *et al.* (4) were downloaded from GEO (series GSE3158). Probes, uniquely and differentially regulated, in an oncogenic HMEC model were calculated between the given pathway and all other samples: both green fluorescent protein (GFP) control and transfected oncogenic HMEC samples. Methods to determine differential expression were consistent with those applied to our data, i.e. permutation testing (500 permutations), a minimum fold-change of 1.5, and a FDR less than 1%. Experimental pathway regulation was calculated using similar methods as those applied to estimate canonical pathway expression, except pathway induced genes increased the pathway metric and inhibited genes decreased the metric. Specifically, probe-level data were first normalized (Z-score), pathway induced probes were summed, pathway inhibited probes were subtracted, and the total was divided by the number of pathway probes available on the given platform. Data across platforms were mapped by Entrez gene identifiers. Differential pathway regulation was assessed by permutation testing (permutations = 500, FDR < 1%) and a p-value with Bonferroni's correction applied.

LWS Data

The LWS-81 gene signature determined by treating PC9 and H2030 lung cancer cells with Wnt3A and published by Nguyen *et al*. (5) was matched across platforms by Entrez gene identifier. Probes with increased expression after treatment with Wnt3A were deemed Wnt+, and those with negative expression were denoted Wnt-. Pathway metrics were built using the same method applied to the Bild *et al*. oncogenic pathways: this includes normalization, summing Wnt+ probes, subtracting Wnt- probes and dividing by the total number of probes. Differential analysis was conducted using consistent methods. Significance was determined by p-value with Bonferroni's correction applied in combination with permutation testing.

Wnt/β-catenin Classifier

The Wnt classifier was built using a closest shrunken centroid approach implemented in R/Bioconductor (2) using the package "pamr" (6). To aptly characterize Wnt/β-catenin signaling we trained a classifier to identify β-catenin signaling as compared to both normal (GFP) and other oncogenic signaling (E2F3, Myc, Ras, and Src) using the adenoviral vector transformed HMEC data published by Bild *et al.* (4). Feature selection was based on a threshold yielding a FDR of 1% or less determined using the function "pamr.fdr" and 500 permutations. In cross validation the model was 100% sensitive, identifying all β-catenin transfected cells, and 97.83% specific, identifying all but one other sample correctly.

Cross Validation of Wnt Classifier

		**Classifier**	
		β-catenin	Other
**Actual**	β-catenin	9	0
	Other	1	45

This classifier was applied to the meta-analysis of 11 studies covering 1,878 primary tumor expression profiles (7-15). Application of the classifier to Affymetrix, Agilent, and Illumina data sets, followed the recommendations of pamr software (6). Specifically, data was consistently normalized within Affymetrix, Agilent, and Illumina platforms. In cross platform comparisons probes were mean averaged to gene level, samples were normalized, feature selection was limited those available on the given platform. The classifier retained the same cross-validation sensitivity and specificity regardless of platform. Multiple experiments within platforms were batch adjusted using the "pamr.batchadjust" R function of the "pamr" package.

Meta-analysis Data Sets

Data for breast cancer meta-analyses (7-16) were downloaded from Gene Expression Omnibus (GEO; http://www.ncbi.nlm.nih.gov/geo/) (17). CEL files for Affymetrix HG-U133A chips (GEO platform: GPL96) were loaded into Expression Console and MAS 5.0 normalized with a target intensity of 600 and exported in a linear format. Agilent data was downloaded using the normalized series matrix files for the UNC custom array (GEO platform: GPL1390) and duplicate samples between the UNNCH studies were removed. Additionally, samples with a degraded RNA profile, as identified by the authors since publication (https://genome.unc.edu/), were also removed. Clinical data for the UNCCH studies were ascertained from the GEO deposits. Metastasis data for the EMC-286, EMC-192, and MSKCC-99 data sets are those published by Bos *et al*. (8).

Meta-Analysis Data Sets

Data Set	GEO	PMID	Size	Platform	Clinical & Outcome Data										
					Intrinsic Subtype	ER	PR	HER2	Grade	Node	Chemo Treatment	Hormone Treatment	Relapse	Mets	OS
**EMC-192**	GSE12276	19421193	192	Affy	N	N	N	N	N	N	Y	Y	N	Y	N
**EMC-286**	GSE2034	15721472	286	Affy	N	Y	N	N	N	Y	Y	Y	N	Y	N
**Georgia**	GSE18539		143	Illm	N	Y	Y	Y	N	N	N	N	N	N	Y
**GGI-327**	GSE6532	17401012	327	Affy	N	Y	Y	N	Y	Y	Y	Y	Y	Y	N
**MSKCC-99**	GSE2603	16049480	99	Affy	N	Y	Y	Y	Y	Y	N	N	N	Y	N
**Quebec**	GSE17650		97	Illm	N	Y	Y	Y	N	N	N	N	N	N	N
**STH-159**	GSE1456	16280042	159	Affy	Y	N	N	N	Y	N	Y	N	Y	N	Y
**UNCCH-186**	GSE10886	19204204	186	Agil	Y	N	N	N	Y	Y	N	PA	Y	N	Y
**UNCCH-67**	GSE6128	17663798	67	Agil	Y	Y	N	N	Y	Y	N	PA	Y	N	Y
**UNCCH-73**	GSE3165	19291283	73	Agil	Y	Y	N	N	Y	Y	N	PA	Y	N	Y
**Uppsala-249**	GSE4922	17079448	249	Affy	N	Y	N	N	Y	Y	N	Y	Y	N	N

PA: partial data

Analysis of Classifier Data

Patients classified as Wnt+ and Wnt- were analyzed for overrepresentation in pathological determined subtypes, intrinsic subtypes, histological grade, and lymph node status using Fisher's exact test implemented in R/bioconductor (2) using the function "fisher.test" of the stats package. A representative example of a two by two table is provided below.

**TN Subtype / Classifier**	**Wnt+**	**Wnt-**	**Total**	
**TN**	52	103	155	**P = $6.3x10^{-14}$**
**non-TN**	4	151	155	
**Total**	56	254	310	

Kaplan-Meier analyses were created in R/Bioconductor (2) using the "survfit" function of the "survival" package. Significant differences in survival were calculated using a log-rank test p-value implemented in R/Bioconductor using the "survdiff" function of the "survival" package.

Reference List

1. Tusher VG, Tibshirani R, & Chu G (2001) Significance analysis of microarrays applied to the ionizing radiation response. *Proc Natl Acad Sci U S A* 98(9):5116-5121.
2. Reimers M & Carey VJ (2006) Bioconductor: an open source framework for bioinformatics and computational biology. *Methods Enzymol* 411:119-134.
3. Kanehisa M (2002) The KEGG database. *Novartis Found Symp* 247:91-101; discussion 101-103, 119-128, 244-152.
4. Bild AH*, et al.* (2006) Oncogenic pathway signatures in human cancers as a guide to targeted therapies. *Nature* 439(7074):353-357.
5. Nguyen DX*, et al.* (2009) WNT/TCF signaling through LEF1 and HOXB9 mediates lung adenocarcinoma metastasis. *Cell* 138(1):51-62.
6. Tibshirani R, Hastie T, Narasimhan B, & Chu G (2002) Diagnosis of multiple cancer types by shrunken centroids of gene expression. *Proc Natl Acad Sci U S A* 99(10):6567-6572.
7. Wang Y*, et al.* (2005) Gene-expression profiles to predict distant metastasis of lymph-node-negative primary breast cancer. *Lancet* 365(9460):671-679.
8. Bos PD*, et al.* (2009) Genes that mediate breast cancer metastasis to the brain. *Nature* 459(7249):1005-1009.
9. Minn AJ*, et al.* (2005) Genes that mediate breast cancer metastasis to lung. *Nature* 436(7050):518-524.
10. Parker JS*, et al.* (2009) Supervised risk predictor of breast cancer based on intrinsic subtypes. *J Clin Oncol* 27(8):1160-1167.
11. Hoadley KA*, et al.* (2007) EGFR associated expression profiles vary with breast tumor subtype. *BMC Genomics* 8:258.
12. Hu Z*, et al.* (2009) A compact VEGF signature associated with distant metastases and poor outcomes. *BMC Med* 7:9.
13. Pawitan Y*, et al.* (2005) Gene expression profiling spares early breast cancer patients from adjuvant therapy: derived and validated in two population-based cohorts. *Breast Cancer Res* 7(6):R953-964.
14. Loi S*, et al.* (2007) Definition of clinically distinct molecular subtypes in estrogen receptor-positive breast carcinomas through genomic grade. *J Clin Oncol* 25(10):1239-1246.
15. Ivshina AV*, et al.* (2006) Genetic reclassification of histologic grade delineates new clinical subtypes of breast cancer. *Cancer Res* 66(21):10292-10301.
16. Herschkowitz JI*, et al.* (2007) Identification of conserved gene expression features between murine mammary carcinoma models and human breast tumors. *Genome Biol* 8(5):R76.
17. Edgar R, Domrachev M, & Lash AE (2002) Gene Expression Omnibus: NCBI gene expression and hybridization array data repository. *Nucleic Acids Res* 30(1):207-210.
